# Hydrophobic alkyl chains substituted to the 8-position of cyclic nucleotides enhance activation of CNG and HCN channels by an intricate enthalpy - entropy compensation

**DOI:** 10.1038/s41598-018-33050-5

**Published:** 2018-10-08

**Authors:** Maik Otte, Andrea Schweinitz, Michele Bonus, Uta Enke, Christina Schumann, Holger Gohlke, Klaus Benndorf

**Affiliations:** 1 Institut für Physiologie II, Universitätsklinikum Jena, Friedrich-Schiller-Universität Jena, 07740 Jena, Germany; 2 Institut für Pharmazeutische und Medizinische Chemie, Heinrich-Heine-Universität Düsseldorf, 40225 Düsseldorf, Germany; 3 Fachbereich Medizintechnik und Biotechnologie, Ernst-Abbe-Hochschule Jena, 07745 Jena, Germany; 4 John von Neumann Institute for Computing (NIC), Jülich Supercomputing Centre (JSC) & Institute for Complex Systems - Structural Biochemistry (ICS 6), Forschungszentrum Jülich GmbH, Jülich, Germany

## Abstract

Cyclic nucleotide-gated (CNG) and hyperpolarization-activated cyclic nucleotide-gated (HCN) channels are tetrameric non-specific cation channels in the plasma membrane that are activated by either cAMP or cGMP binding to specific binding domains incorporated in each subunit. Typical apparent affinities of these channels for these cyclic nucleotides range from several hundred nanomolar to tens of micromolar. Here we synthesized and characterized novel cAMP and cGMP derivatives by substituting either hydrophobic alkyl chains or similar-sized more hydrophilic heteroalkyl chains to the 8-position of the purine ring with the aim to obtain full agonists of higher potency. The compounds were tested in homotetrameric CNGA2, heterotetrameric CNGA2:CNGA4:CNGB1b and homotetrameric HCN2 channels. We show that nearly all compounds are full agonists and that longer alkyl chains systematically increase the apparent affinity, at the best more than 30 times. The effects are stronger in CNG than HCN2 channels which, however, are constitutively more sensitive to cAMP. Kinetic analyses reveal that the off-rate is significantly slowed by the hydrophobic alkyl chains. Molecular dynamics simulations and free energy calculations suggest that an intricate enthalpy - entropy compensation underlies the higher apparent affinity of the derivatives with the longer alkyl chains, which is shown to result from a reduced loss of configurational entropy upon binding.

## Introduction

Cyclic nucleotide-gated (CNG) and hyperpolarization-activated cyclic nucleotide-gated (HCN) channels are activated by the binding of cyclic nucleotide monophosphates (cNMPs) to specific intracellular cyclic nucleotide binding domains (CNBDs)^1–3^. Despite this functional similarity, the physiological role and activation mechanism of both channel types are markedly different: CNG channels generate receptor potentials in photoreceptors and olfactory sensory neurons^4,5^ and are solely activated by the binding of cNMPs. In contrast, HCN channels are primarily activated by hyperpolarizing voltage and activation is secondarily enhanced by the binding of cAMP^2,3,6–10^. HCN channels generate electrical rhythmicity in specialized cells of the heart and brain. In brain neurons they also contribute to setting the membrane potential and to dampen arriving inhibitory and excitatory stimuli (for review see Biel and coworkers^11^). Both channel types are nonspecific cation channels and, concerning their topology, they belong to the superfamily of six-transmembrane domain voltage-gated channels^12^: An intracellular N-terminus is followed by the α-helical transmembrane domains S1 to S6, with S4 being the voltage sensor, and a hairpin loop between S5 and S6 dipping into the membrane, thereby contributing to the wall of the common pore. In contrast to solely voltage-gated channels, the C-terminus in each subunit contains the CNBD that is connected to the S6 helix via the C-linker.

Native mammalian olfactory CNG channels are composed of three different subunit isoforms, 2xCNGA2, CNGB4 and CNGB1b, of which only CNGA2 subunits can form functional homotetrameric channels when expressed heterologously^4^. For homologous TAX-4 channels of *Caenorhabditis elegans*, a full channel structure at 3.5 Å resolution has been reported recently^13^. Mammalian HCN channels are either homo- or heterotetrameric channels composed of the four subunit isoforms HCN1 to HCN4^14,15^. All four isoforms can form functional homotetrameric channels. Among the different homotetrameric channels HCN2 channels are strongly modulated by cAMP^16^. The binding of cAMP shifts voltage-dependent activation to more depolarized potentials and increases both the rate of channel opening and the maximal current at saturating hyperpolarizing voltages^17^. Recently, the structure of the full length HCN1 isoform has been published by means of cryo-electron microscopy at 3.5 Å resolution both in the absence and presence of cAMP^18^. Notably, the structure of the tetrameric CNBD of the full-length HCN1 channel is closely similar to respective structures of the isolated CNBDs from three mammalian HCN isoforms^19–21^.

Based on this structure a scenario for the duality of voltage- and cAMP-induced activation has been proposed. The cAMP-effect to activate the channels has been explained by inducing a concerted rotation of the cytoplasmic domains enhancing opening of the gate formed by the S6-helices in the sense of a relief of auto-inhibition^22,23^. In more detail, the molecular mechanism of cNMP binding to the CNBD is complex. The CNBD consists of the helices A to C and a β-roll, in which a phosphate binding cassette is embedded, which in turn contains a further short α-helix termed P-helix. For the CNBD of HCN channels, cAMP has been reported to bind in its *anti* conformation^19^. The purine ring interacts with the C-helix by a hydrophobic interaction (I636) and by the N6 amine forming a hydrogen bond with backbone carbonyl oxygen of R632. The purine ring interacts via hydrophobic interactions with V564, M572, L574 in the β-roll. Furthermore, the phosphate is stabilized by three hydrogen bonds and an ionic interaction at the phosphate binding cassette. In contrast to cAMP, cGMP binds in the *syn* conformation, enabling its purine ring N2 atom to form a hydrogen bond with T592 of the β-roll^19^.

In the past decades, several studies addressed the effects of cNMP modifications on the activation of CNG channels^24,25^, HCN channels^26^ and isolated CNBDs of HCN channels^27–29^. A major result is that an enhanced apparent affinity can be reached by 8-thio-substituted compounds^30^, suggesting sizeable space around the 8-position of the purine ring of a bound cNMP. Even bulky substituents such as 8-pCPT-, 8-Fluo- or 8-NBD-cNMP can be accommodated^26,31–33^.

Herein we systematically investigated the impact of modifications at the 8-position of the purine base by thioalkylamines or thioheteroalkylamines within a set of 20 cGMP- and cAMP-derived ligands and quantified their agonistic effects on functional channels. This approach provides the advantage that all cooperative effects among the four CNBDs^34,35^ and reciprocal interactions with the channel core^36^ are included. To enable more general conclusions, we analyzed three types of channels: homotetrameric CNGA2 channels, heterotetrameric CNGA2:CNGA4:CNGB1b channels and homotetrameric HCN2 channels. For a subset of cNMP derivatives, molecular docking and simulations as well as end-point free energy computations were performed to reveal the underlying reasons for the observed structure-apparent affinity relationships.

## Results

### Cyclic nucleotide derivatives

We systematically modified the 8-position of the cNMPs by thioalkylamine residues of increasing length including an ethyl, hexyl or decyl chain. In general an increasing length of the alkyl chain introduces stronger hydrophobicity. To probe the effects of charged versus uncharged residues, N-terminal acetylated and non-acetylated compounds were synthesized. In addition, similar-sized more hydrophilic thioheteroalkylamine residues with different hetero atoms were attached to the 8-position. These include a short polyethylene glycol, and glycine. Names, structures and short names, as used in the text, are summarized in Table 1. The synthesis strategies of the cNMP derivatives are described in the Methods section.

**Table 1:**
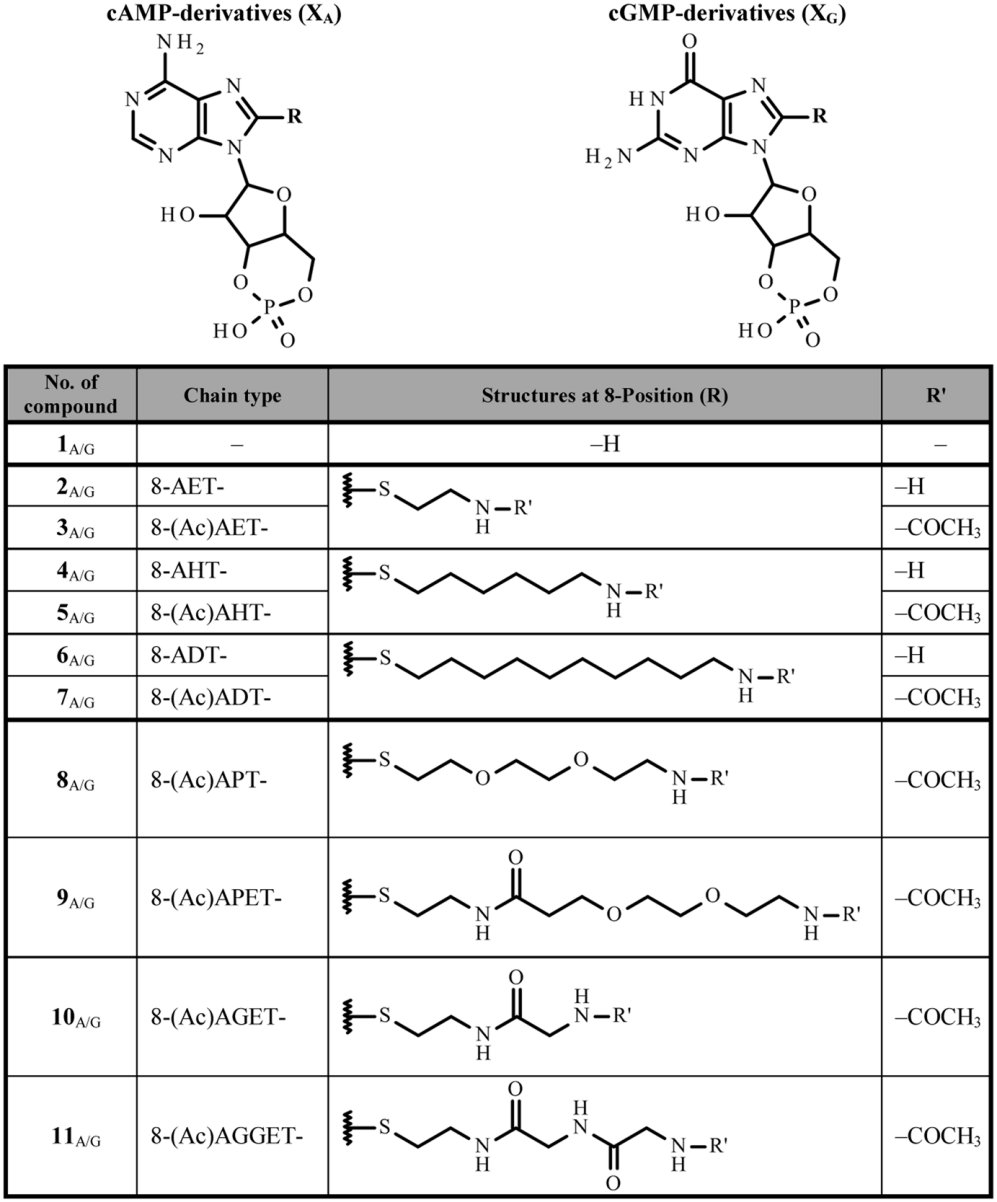
Names and structures of cyclic nucleotide derivatives used in the experiments. cNMP is either cGMP (X_G_) or cAMP (X_A_).

### Hydrophobic moieties in 8-position of cyclic nucleotides enhance the apparent affinity to CNG channels

**Figure 1:**
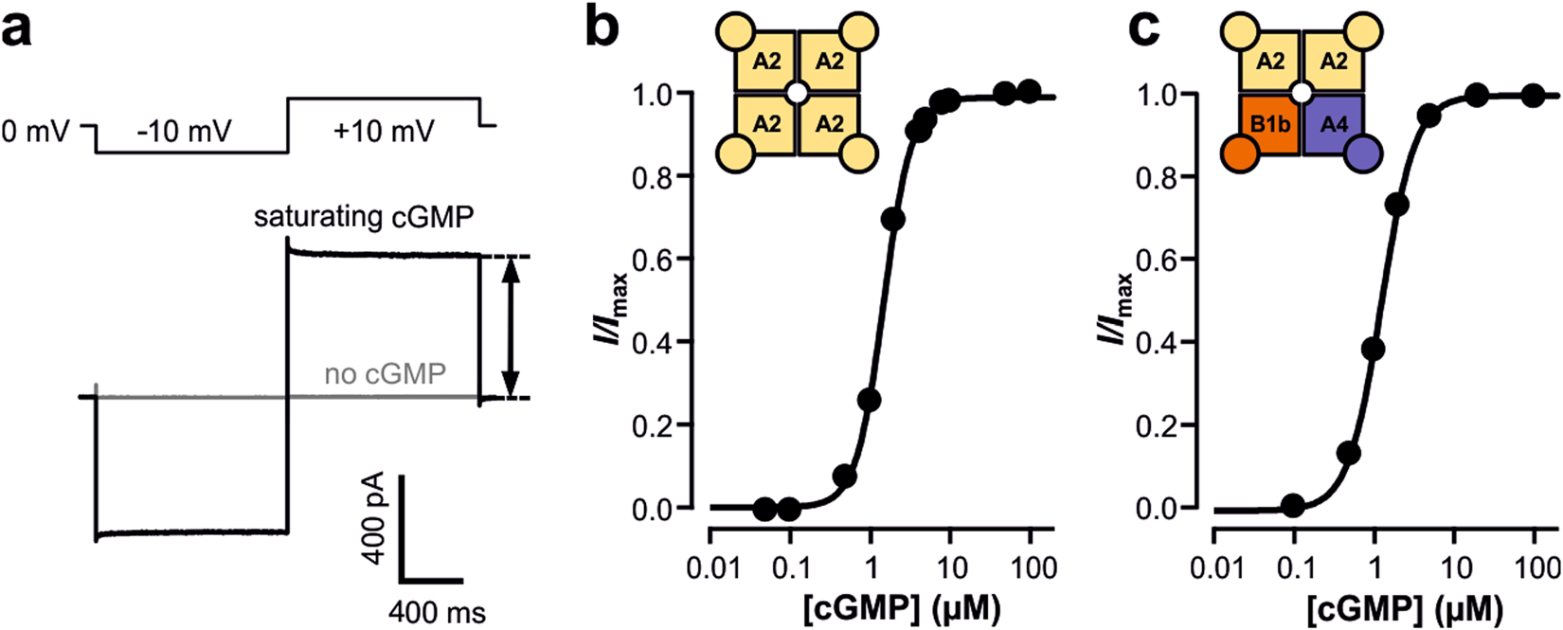
Current measurement and concentration-activation relationships in olfactory CNG channels. (**a**) Representative current recording from CNGA2 channels activated by saturating cGMP (**1**_G_, 100 µM) according to the indicated voltage protocol. The amplitude of the late current at +10 mV was evaluated for the concentration-activation relationships (black arrow). (**b**) Concentration-activation relationship for CNGA2 channels. The continuous curve was obtained by fitting equation (1) yielding *EC*_50_ = 1.47 µM and H = 2.52. (**c**) Concentration-activation relationship for heterotetrameric CNGA2:CNGA4:CNGB1b channels. *EC*_50_ = 1.24 µM and H = 2.09.

Ionic currents of both homotetrameric CNGA2 and heterotetrameric CNGA2:CNGA4:CNGB1b channels incorporated in inside-out patches were elicited by applying voltage pulses to −10 and +10 mV, starting from a holding potential of 0 mV (Fig. 1a). The current amplitude was generally measured as the late current amplitude at +10 mV with respect to the current amplitude in the absence of the cNMP, which was only negligibly small. For each cNMP derivative a complete concentration-activation relationship was recorded, and the data points were fitted with the Hill equation (equation 1) yielding the concentration of half maximum activation (*EC*_50_) and the Hill coefficient *H* (Fig. 1b,c).

**Figure 2:**
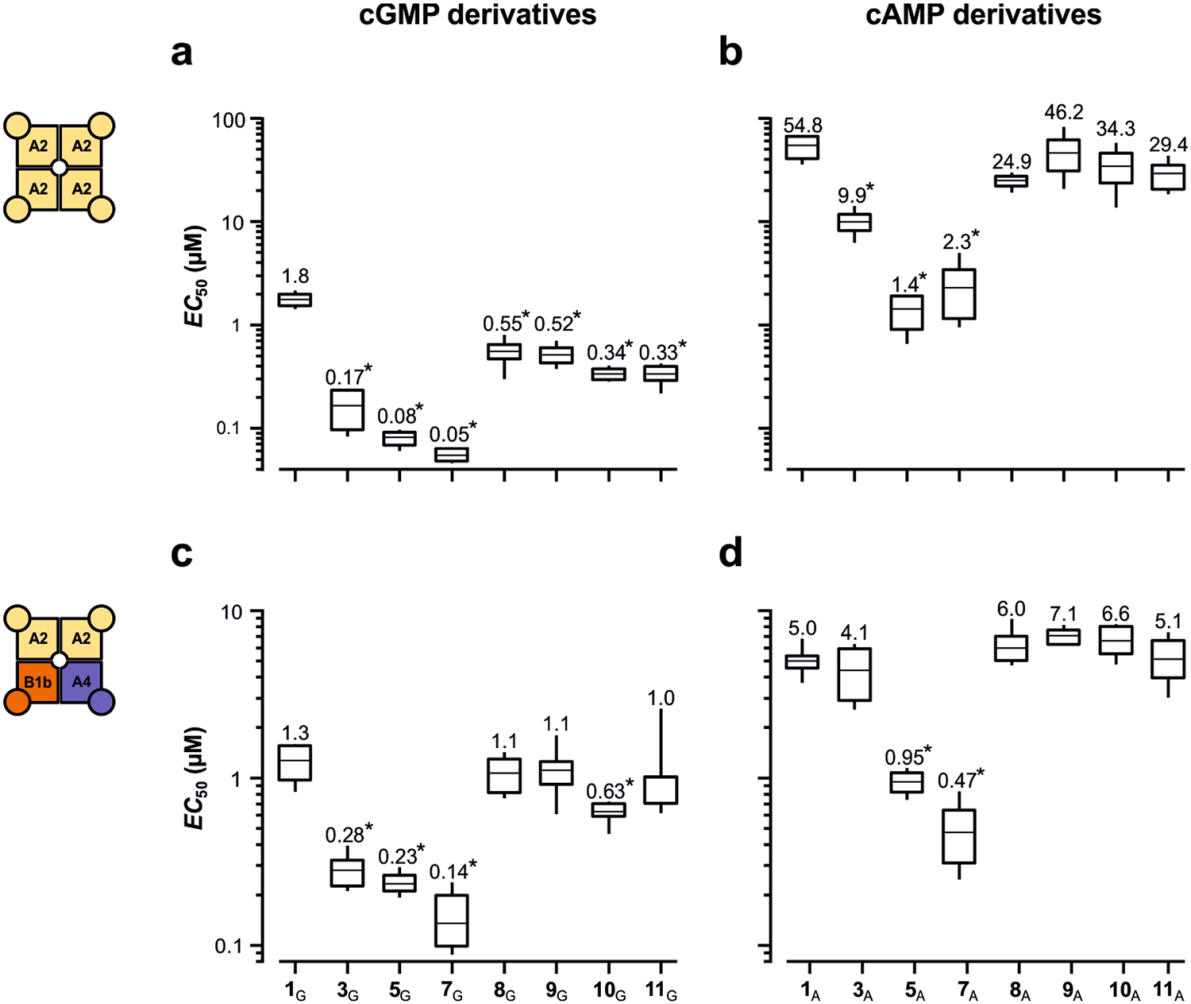
Box plot of the effect of cNMP derivatives on the *EC*_50_ value for CNG channels. Shown are mean as a horizontal line within each box, the boxes as 25^th^ and 75^th^ percentiles and the whiskers as 10^th^ and 90^th^ percentiles of the data. The numeric mean values are indicated above each box. The asterisks indicate *EC*_50_ values that were significantly smaller than the respective natural cyclic nucleotide **1**_G/A_ (*p* < 0.01). (**a**) CNGA2 channels with cGMP derivatives. (**b**) CNGA2 channels with cAMP derivatives. (**c**) CNGA2:CNGA4:CNGB1b channels with cGMP derivatives. (**d**) CNGA2:CNGA4:CNGB1b channels with cAMP derivatives. Independent of the channel type and the cNMP, the most hydrophobic residues produce the highest apparent affinity whereas the hydrophilic residues, though similar by size, leave the apparent affinity approximately unaffected.

We first determined the effect of alkyl chains with increasing lengths on CNGA2 and CNGA2:CNGA4:CNGB1b channels (Fig. 2). First consider the effects of the cGMP derivatives (Fig. 2a,c). Compared to cGMP (**1**_G_), 8-(Ac)AET-cGMP (**3**_G_) reduced the *EC*_50_ value significantly, and this effect was further enhanced by 8-(Ac)AHT-cGMP (**5**_G_). Prolongation with a decyl chain in 8-(Ac)ADT-cGMP (**7**_G_) did not further reduce the *EC*_50_ value. In contrast, respectively sized more hydrophilic residues (compounds **8**_G_–**11**_G_) left the *EC*_50_ value approximately unaffected compared to **1**_G_.

Next consider the effects of the respective cAMP derivatives (Fig. 2b,d). Notably, the pattern produced by the cGMP derivatives was largely reproduced by the respective cAMP derivatives with the only difference that all *EC*_50_ values were shifted towards higher values. When considering the Hill coefficient *H* for the respective cGMP and cAMP derivatives, a systematic change at prolonged hydrophobic chains could not be observed (Supplementary Tables 1a–e).

Together, the tested cGMP and cAMP derivatives produce a consistent activity pattern on both homo- and heterotetrameric CNG channels. Moreover, the results allowed us to identify highly potent agonistic cGMP derivatives for homotetrameric CNGA2 channels with *EC*_50_ values in the range of tens of nanomolar.

### The effect of the length of the alkyl chain on the apparent affinity dominates over the effect of a terminal charge

**Figure 3:**
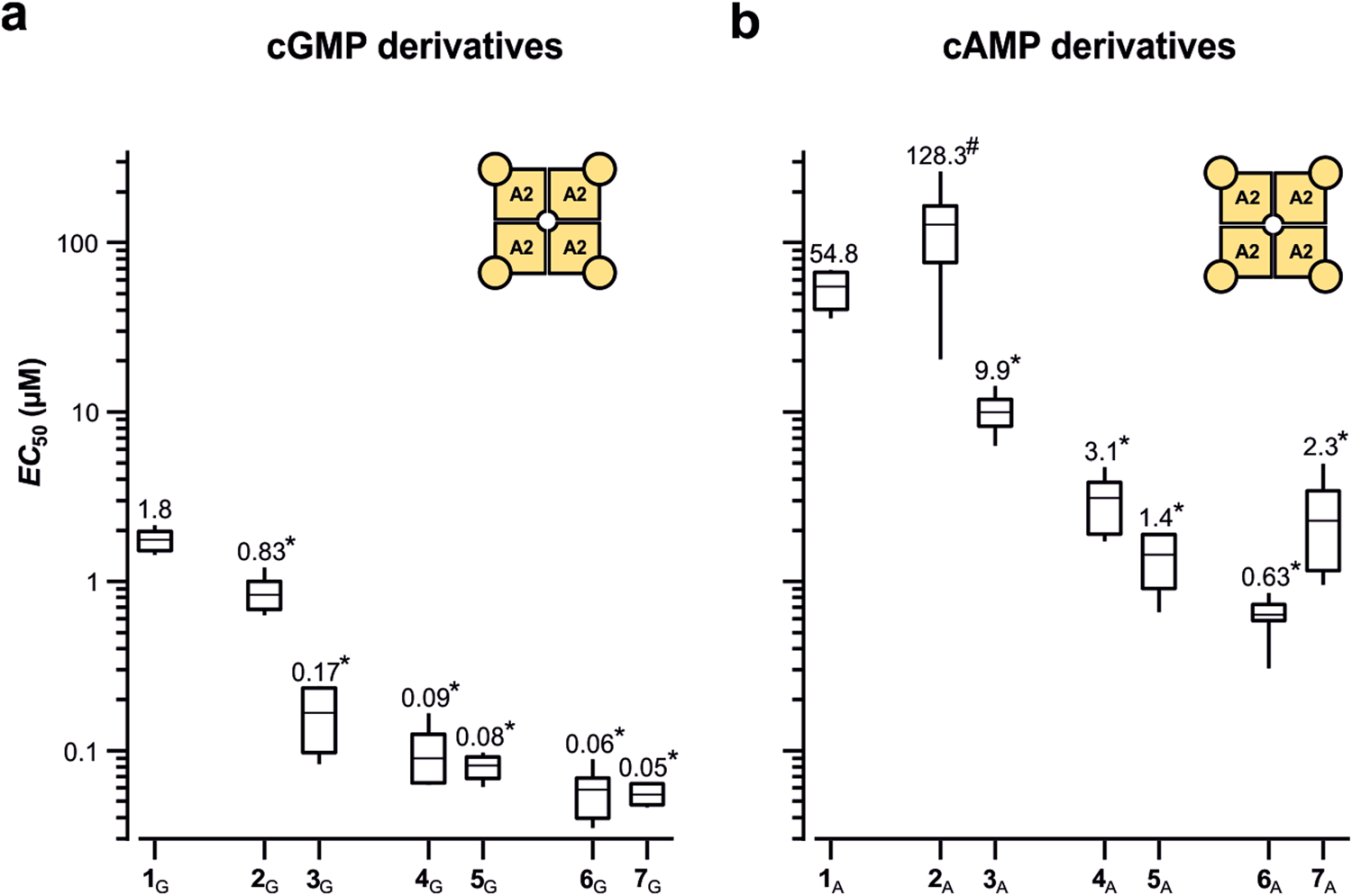
Comparison of the effects of N-acetylated with non-acetylated thioalkylamine residues in CNGA2 channels. Box plot of the *EC*_50_ values. Generally, the longer residues cause a lower *EC*_50_ value (c.f. Supplementary Table 1a,b). The *EC*_50_ values of all cGMP and cAMP derivatives were significantly smaller (asterisks) than those of the natural cyclic nucleotides **1**_G_ and **1**_A_, respectively (*p* < 0.01). The “#” indicates that compound **2**_A_ is a partial agonist.

So far, we showed that a thioalkylamino chain in 8-position of the cNMPs enhanced the apparent affinity for cNMPs and that six C-atoms apparently suffice to generate the maximum effect. To test whether the observed effects are influenced by a terminal charge at the chain, we compared the effects of N-acetylated with non-acetylated chains, i.e., neutral with positively charged chains. For the cGMP derivatives with an ethyl chain the acetyl group reduced the *EC*_50_ value about five times (0.83 µM for **2**_G_ versus 0.17 µM for **3**_G_) in homotetrameric CNGA2 channels. However, with a hexyl or decyl chain, this effect vanished, suggesting that the total length of the chain is the dominating influence on the *EC*_50_ value (Fig. 3a), while the charge at the terminus of the chain is rather irrelevant. For the cAMP derivatives the pattern is approximately preserved (Fig. 3b). Concerning the ethyl chain, the acetyl group reduced the *EC*_50_ value about 12 times compared to the non-acetylated chain (128.3 µM for **2**_A_ versus 9.9 µM for **3**_A_), though it should be noted that the derivative **2**_A_ is exceptionally only a partial agonist. Prolongation of the chain increases the apparent affinity further, independent of the charge (derivatives **4**_A_**–7**_A_ in Fig. 3b).

### In CNG channels the hydrophobic residues in 8-position predominantly slow down the unbinding rate

To learn more about the molecular mechanism of the enhanced apparent activity by alkyl chains in 8-position of cGMP and cAMP, we analyzed for a representative highly potent cGMP derivative the activation and deactivation time course. We applied concentration jumps from zero to a defined level and back to zero, yielding the time constants *τ*_a_ and *τ*_d_, respectively (c.f. Methods section). If the enhancement of the apparent affinity is caused by solely accelerating the binding rate, *τ*_a_ would be decreased and *τ*_d_ would be unaffected. Conversely, if the hydrophobic alkyl chain enhances the apparent affinity by solely decelerating the unbinding rate, *τ*_d_ would be increased and *τ*_a_ could be increased preferentially at lower concentrations.

**Figure 4:**
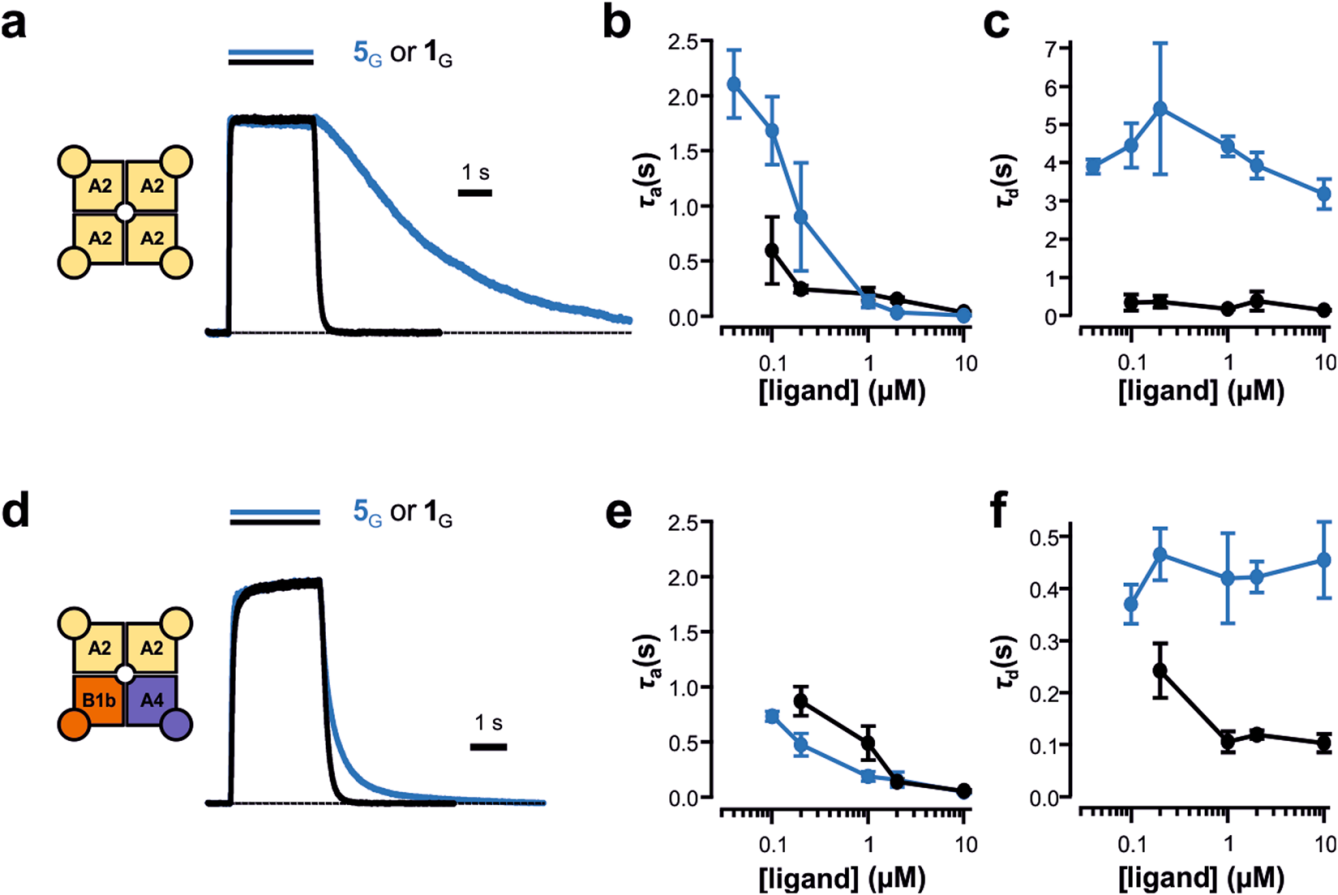
The hydrophobic residue in 8-position slows down the unbinding of 8-(Ac)AHT-cGMP (**5**_G_) compared to cGMP (**1**_G_). The channels were activated and deactivated by concentration jumps evoked by a piezo actuator switching between a control solution and a test solution containing a defined ligand concentration. (**a**) Superimposition of a current time course at 10 µM of **5**_G_ and **1**_G_ in CNGA2 channels. (**b**) Activation time constant *τ*_a_ as function of the **5**_G_ and **1**_G_ concentration in CNGA2 channels. (**c**) Deactivation time constant *τ*_d_ as function of the **5**_G_ and **1**_G_ concentration in CNGA2 channels. (**d**,**e**,**f**) Analog to a, b, c for CNGA2:CNGA4:CNGB1b channels. Error bars indicate SEM.

We chose 8-(Ac)AHT-cGMP (**5**_G_) as representative cGMP derivative and tested it with both CNGA2 and CNGA2:CNGA4:CNGB1b channels. With CNGA2 channels (10 µM cGMP) the activation time course was rather unaffected (Fig. 4a), whereas deactivation was markedly slowed. The plot of the activation time constant *τ*_a_ as function of the ligand concentration showed that at 1 µM 8-(Ac)AHT-cGMP (**5**_G_) and higher concentrations, τ_a_ was similar to that for cGMP (**1**_G_), whereas it was larger at lower concentrations (Fig. 4b). In contrast, the deactivation time constant *τ*_d_ was approximately an order of magnitude increased at all tested concentrations of **5**_G_ (Fig. 4c).

With CNGA2:CNGA4:CNGB1b channels, the effects of 10 µM **5**_G_ on activation and deactivation kinetics were qualitatively similar to those with CNGA2 channels, with the difference that deactivation was slowed to a much lower degree (Fig. 4d–f). This reduced effect on deactivation also caused that *τ*_a_ was not affected at low concentrations of **5**_G_ (Fig. 4e). Together these results suggest that the hydrophobic chain in 8-position specifically decreases the unbinding rate in both types of channels, which would increase the dwell time in the CNBD. Moreover, the smaller effect of **5**_G_ on CNGA2:CNGA4:CNGB1b channels suggests that the dwell time in the CNGA4 and/or the CNGB1b subunit is shorter than that in the CNGA2 subunits.

### A hydrophobic residue in 8-position of cAMP (**1**_A_) also enhances the apparent affinity to HCN2 pacemaker channels

**Figure 5:**
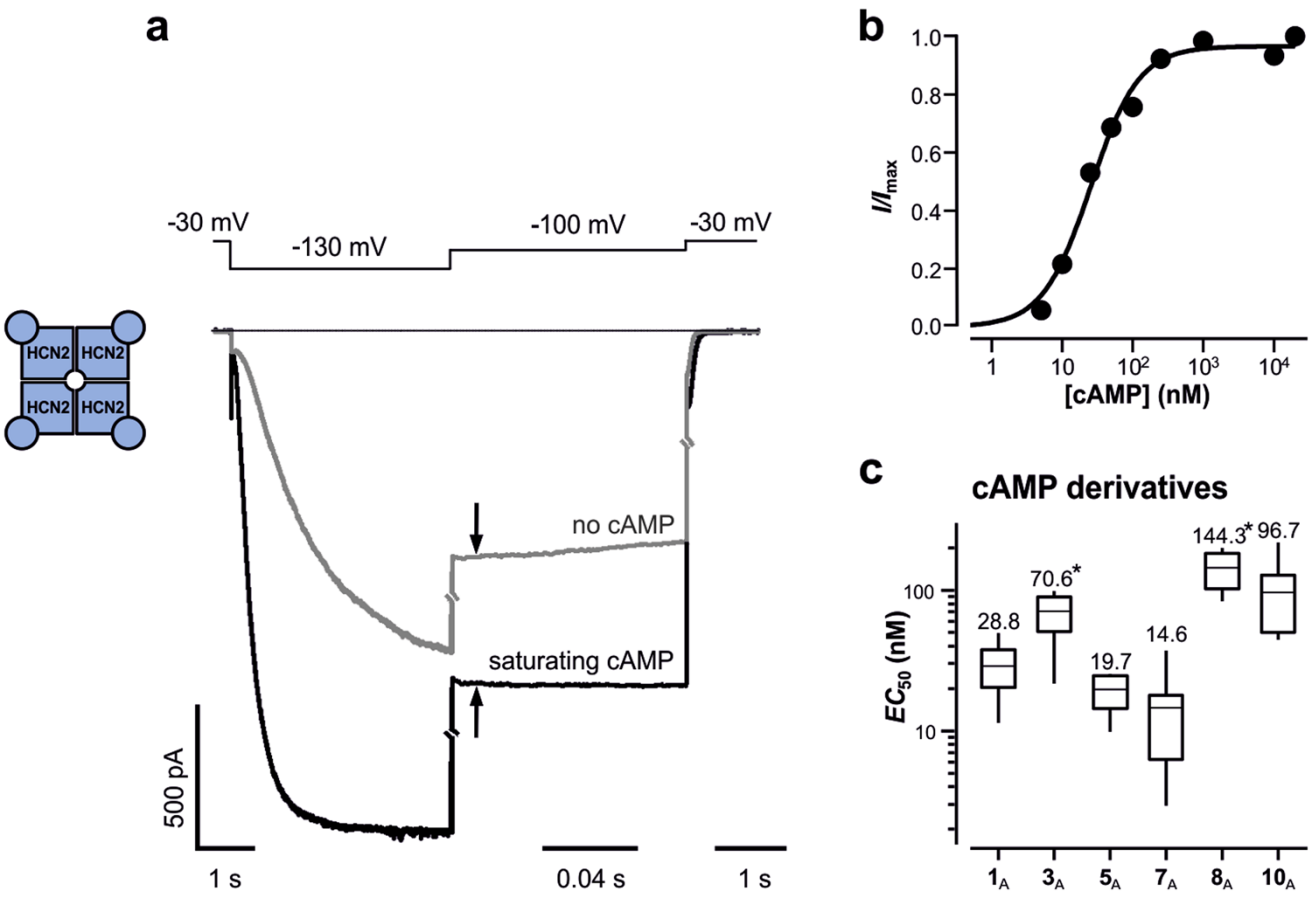
Effects of cAMP derivatives on HCN2 channels. (**a**) Current traces in the absence and presence of saturating cAMP (20 µM) evoked by the indicated pulse scheme. The increase of the instantaneous current at 130 mV by cAMP (arrows) was evaluated. (**b**) Concentration-activation relationship for the cAMP-induced current increase of an individual experiment. The continuous curve was obtained by fitting the Hill equation (equation (1)) yielding *EC*_50_ = 25.42 nM and *H = *1.28. (**c**) Box plot of the *EC*_50_ values for the indicated cAMP derivatives. Shown are mean as a horizontal line within each box, the boxes as 25^th^ and 75^th^ percentiles and the whiskers as 10^th^ and 90^th^ percentiles of the data. The numeric mean values are indicated above each box. The *EC*_50_ values were not significantly different from **1**_A_ apart from **3**_A_ and **8**_A_ which were significantly larger (asterisks; *p* < 0.01).

We next tested whether related HCN2 channels are similarly affected by a hydrophobic chain in 8-position of the purine ring using the natural ligand cAMP (**1**_A_). From a holding potential of 0 mV the channels were pre-activated by hyperpolarizing pulses to −130 mV, and the current amplitude was measured as instantaneous current at −100 mV in both the absence and presence of **1**_A_ (Fig. 5a) and a series of cAMP derivatives. The amplitude of the current differences evoked by **1**_A_ and its derivatives were plotted as function of the concentration, and the data points were fitted by equation 1, yielding again values for *EC*_50_ and *H* (Fig. 5b). Comparing the *EC*_50_ values reveals the following: First, as known for HCN2 channels, a lower **1**_A_ concentration (28.8 nM) was required for activation than for either **1**_A_ or **1**_G_ on CNG channels (c.f. Fig. 2). Second, **3**_A_ did not decrease but slightly increase the *EC*_50_ value. Third, **5**_A_ and **7**_**A**_ further decreased the *EC*_50_ value but the effects were only moderate. Fourth, the more hydrophilic thioheteroalkyl chains with a PEG (**8**_A_) or a glycine (**10**_A_) increased the *EC*_50_ value about threefold. Nevertheless, the overall pattern for the five tested cAMP derivatives was similar to that for CNG channels: A longer hydrophobic alkyl chain increased the apparent affinity of the derivatives whereas the more hydrophilic heteroalkyl derivatives were ineffective in this respect. The Hill coefficients did not show a systematic dependence on the 8-substitutions (c.f. Supplementary Table 1e).

### Different potencies of cAMP derivatives in CNGA2 channels likely result from differential binding affinities

We intended to provide an explanation at the atomistic level as to the observed structure-apparent affinity relationships of cAMP derivatives (**2**_A_–**7**_A_) shown in Supplementary Fig. 1 with respect to binding to CNGA2 channels. We did not consider the heteroalkyl cAMP derivatives (**8**_A_–**11**_A_), because in these cases the range of p*EC*_50_ values (<0.3 log units) is similar to the accuracy limit of our computations^37^. By molecular docking^38^, we initially predicted binding poses (orientations and conformations) of the respective derivatives in a homology model of the homotetrameric CNGA2 channel, which was based on the cryo-EM structure of TAX-4^13^ as a template (overall sequence identity: ~53%; sequence identity in the binding pocket of cGMP: ~77%).

**Figure 6:**
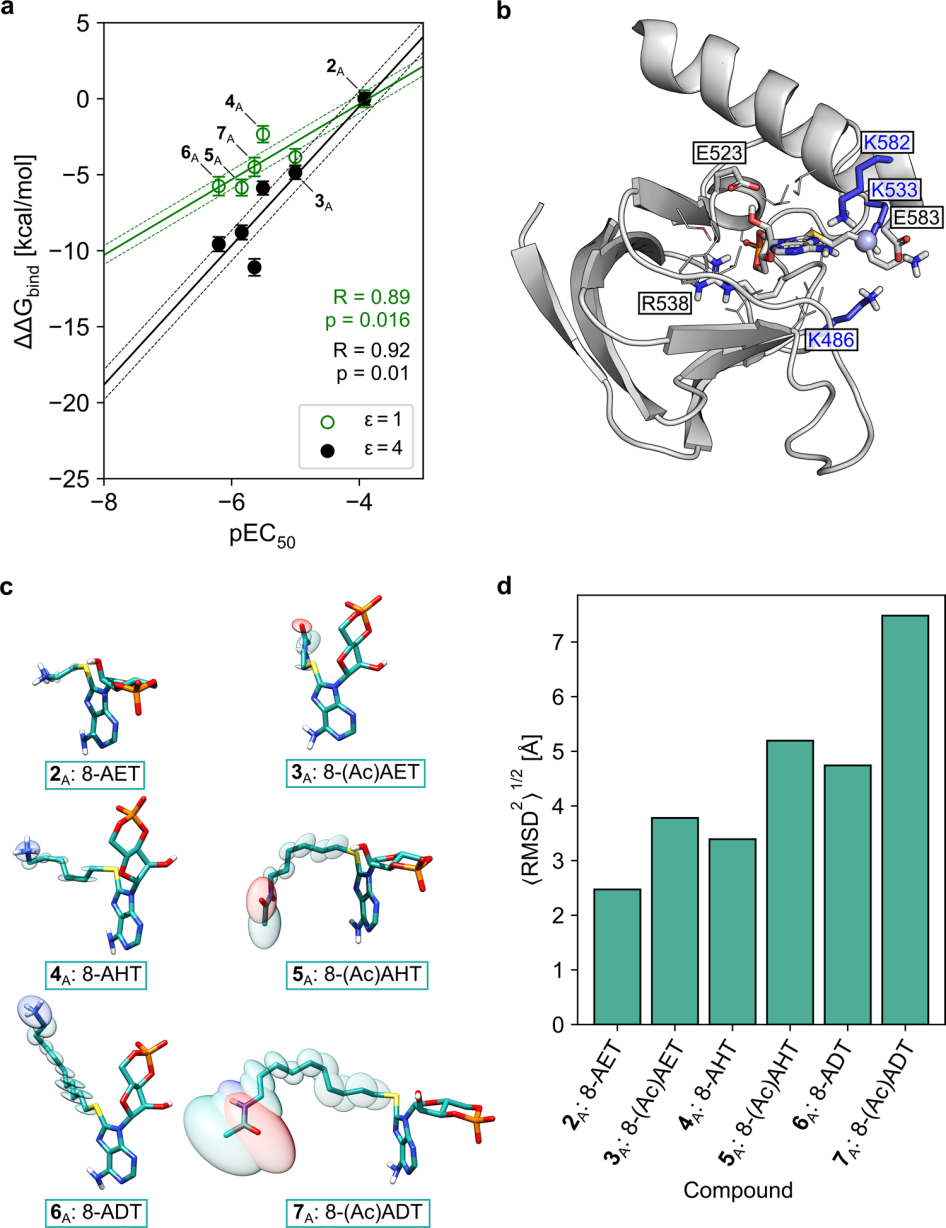
Calculated relative binding free energies (ΔΔ*G*_bind_) and conformational heterogeneity of cAMP derivatives in CNGA2 channels. (**a**) ΔΔ*G*_bind_ with respect to 8-AET-cAMP (**2**_A_) for cAMP derivatives of the congeneric series carrying C_2_-, C_6_-, and C_10_-substituents (**3**_A_–**7**_A_) for internal dielectric constants of ε = 1.0 (green circles) and ε = 4.0 (black, filled circles). Linear regression lines are drawn as solid lines in the respective colors, and the standard error of the estimate is indicated by the surrounding dashed lines. (**b**) Binding pose of 8-AHT-cAMP (4_A_; grey sticks, center) in a single CNBD. Residues within 5 Å of the ligand are depicted explicitly. Residues forming hydrogen bonds and/or electrostatic interactions to the ligand are depicted as sticks, other residues are depicted as lines. Lysines K486, K533 and K582 are colored in blue. A representative position in which the positively charged nitrogen of a C_2_ ligand would be is depicted as blue sphere; the spatial proximity to the surrounding lysines indicates a potential electrostatic repulsion in C_2_ derivatives that opposes binding. (**c**) Visualization of the calculated ADPs (ellipsoid scaling factor: 0.5) for the six cAMP derivatives **2**_A_–**7**_A_. (**d**) Conformational heterogeneity of the tail in the respective cAMP derivatives in the complex with a CNGA2 channel. Bar heights represent the quadratic means of the pairwise root-mean-square deviation (2D-RMSD) of the atomic coordinates of the ligand tail region after root-mean-square fitting of the core/head region.

To further confirm the obtained binding poses, we performed 200 ns of MD simulations for each complex of four cAMP derivatives and a homotetrameric CNGA2 channel in an explicit solvent/explicit membrane environment, followed by MM-PBSA calculations of binding free energies^39^. Prior to that, we derived dihedral parameters for the torsions involving the bond between the carbon at C8 (GAFF atom type *cc*) and the sulfur of the alkylsulfanyl moiety (GAFF atom type *ss*), which show very good agreement with QM-derived torsion energies (see section *Validation of force field parameters* in the Supplementary Results; Supplementary Fig. 2). Although an implementation of MM-PBSA for implicit solvent/implicit membrane calculations of binding free energies is available^40^, the ligand binding site in the intracellular CNBD is far enough away (~45 Å) from the inner leaflet of the membrane to justify omitting an implicit membrane representation in our MM-PBSA computations. All ligands remained stably bound to the cAMP binding site throughout the MD simulations, as evident from an RMSD of the cAMP head group ≤1.5 Å (Supplementary Fig. 3a), though the tails move considerably (Supplementary Fig. 3b), resulting in overall moderate to large chain motions relative to the protein (Supplementary Fig. 3c). The computed binding free energies display a significant and very good correlation with experimentally determined p*EC*_50_ values (Fig. 6a); *R*_ε=1_ = 0.89, *R*_ε=4_ = 0.92), which indirectly supports the quality of the initial binding poses. The standard error of the mean for Δ*G* over all subunits was <0.62 kcal mol^−1^ for all ligands (Fig. 6a), suggesting a precise Δ*G* estimate and only small differences between subunits if all four subunits are ligand-bound. Furthermore, very good correlations were found irrespective of whether the dielectric constant of the protein was set to* ε* = 1 or 4^41^, indicating that the results are robust with respect to this parameter^42^. Our computations demonstrate that binding of the 8-substituted cAMP derivatives **2**_A_–**7**_A_ to CNGA2 channels becomes increasingly favorable with increasing length of the alkyl chain (for *ε* = 1: ΔΔ*G*_C2_ = 0.00 kcal mol^−1^ (reference), ΔΔ*G*_C6_ = −2.33 kcal mol^−1^, ΔΔ*G*_C10_ = −5.75 kcal mol^−1^; the same trend is observed for *ε* = 4) and that removal of the terminal charge via N-acetylation provides the largest gain in affinity for those derivatives carrying an ethyl side chain (for *ε* = 1: ΔΔ*G*_C2_: −3.84 kcal mol^-1^, ΔΔ*G*_C6_: −3.51 kcal mol^−1,^ ΔΔ*G*_C10_: 1.26 kcal mol^−1^; same trend for *ε* = 4), which parallels experimental findings on apparent affinities (c.f. Fig. 3b). Taken together, these results suggest that the measured differences in potency of the cAMP derivatives **2**_A_–**7**_A_ predominantly result from affinity differences at the CNBD itself rather than from differences associated with other parts of the channel.

### Interplay of specific electrostatic interactions and differential configurational entropy losses can explain affinity gains of cAMP derivatives with longer alkyl chains and/or N-acetylation

Analysis of tail motions of cAMP derivatives **2**_A_–**7**_A_ bound to CNGA2 channels in terms of anisotropic displacement parameters (ADP; Fig. 6b,c) reveals rather small ADPs for the derivatives with a terminal charge (**2**_A_, **4**_A_, **6**_A_), which increase only moderately with respect to an increasing length of the alkyl linker. In contrast, for N-acetylated cAMP derivatives (**3**_A_, **5**_A_, **7**_A_), the ADPs are 2.5- to 15-fold larger, and they markedly increase with increasing length of the alkyl linker. Characterizing the conformational heterogeneity of the tail region of the cAMP derivatives in terms of the quadratic means of the pairwise RMSD (<RMSD^2^>^1/2^; Fig. 6d, Supplementary Fig. 4)^43^ reveals a similar picture in that an N-acetylated cAMP derivative shows a larger <RMSD^2^>^1/2^ than the related one with terminal charge, and the <RMSD^2^>^1/2^ values increase more strongly with the length of the alkyl chain for the former than the latter derivatives. These findings point to an intricate contribution of differences in residual tail motions of bound cAMP derivatives **2**_A_–**7**_A_ to differences in the computed binding affinities.

**Figure 7:**
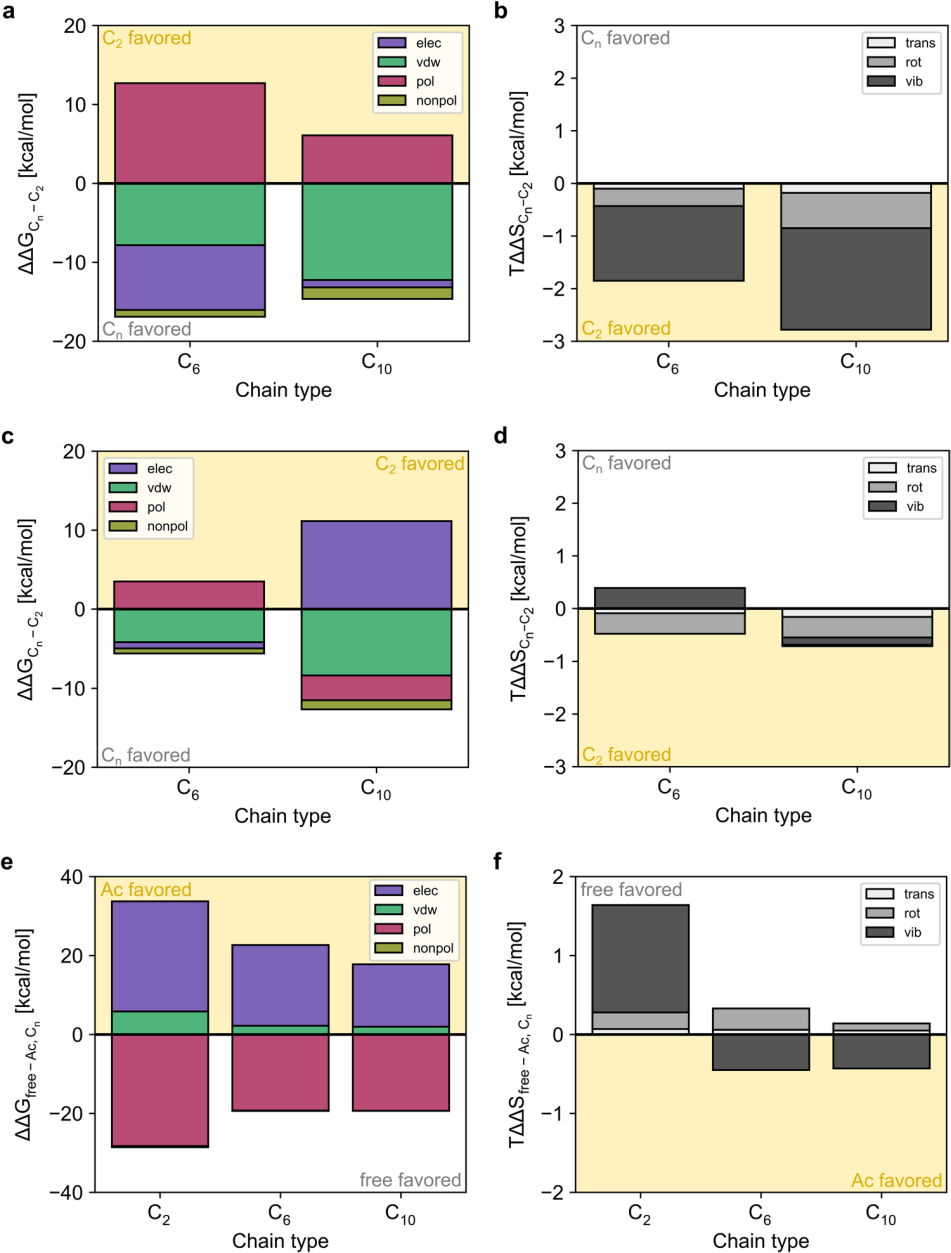
Decomposition of the binding free energy of cAMP derivatives. Binding free energies were decomposed into their respective energy (**a**,**c**,**e**; electrostatic (elec), van der Waals (vdw), polar solvation (pol), and nonpolar solvation (nonpol)) and configurational entropy (**b**,**d**,**f**; translational (trans), rotational (rot), and vibrational (vib)) terms for ε = 1. (**a**,**b**) Difference between 8-AET-cAMP (**2**_A_) and 8-AHT-cAMP (C_6_; **4**_A_) or 8-ADT-cAMP (C_10_; **6**_A_). (**c**,**d**) Difference between 8-(Ac)AET-cAMP (**3**_A_) and 8-(Ac)AHT-cAMP (C_6_; **5**_A_) or 8-(Ac)ADT-cAMP (C_10_; **7**_A_). (**e**,**f**) Difference between 8-AET-cAMP (**2**_A_) and 8-(Ac)AET-cAMP (C_2_; **3 **_A_), 8-AHT-cAMP (**4**_A_) and 8-(Ac)AHT-cAMP (C_6_; **5**_A_), and 8-ADT-cAMP (**6**_A_) and 8-(Ac)ADT-cAMP (C_10_; **7**_A_).

To further scrutinize the role of the tail, we decomposed the computed binding free energies into respective energy and entropy terms (Supplementary eqs 1, 7), as done previously^44^. For both types of cAMP derivatives, either with a terminal charge or N-acetylation, improved van der Waals interactions and non-polar contributions to the solvation free energy foster binding of derivatives with longer alkyl chains (Fig. 7a,c). Electrostatic interactions, counteracted by polar contributions to the solvation free energy, favor binding of longer cAMP derivatives with a terminal charge (Fig. 7a), whereas they disfavor the C_10_ derivative with N-acetylation (Fig. 7c). On a structural level, MD trajectories suggest charge-charge repulsions between short-chain cAMP derivatives with a terminal charge and arginine/lysine residues surrounding the binding site (R635 in HCN2, K486, K533, K582 in rCNGA2; Fig. 6b), which are reduced with increasing chain length. In contrast, in the case of N-acetylated derivatives, a longer alkyl chain abrogates charge-assisted hydrogen bonds between the arginine/lysine residues and the acetyl carbonyl group. Similarly, an increasing length of the alkyl linker leads to different configurational entropy contributions of the cAMP derivatives depending on whether a terminal charge is present or N-acetylation: in the former case, configurational entropy contributions to binding become the more disfavored the longer the alkyl linker is (Fig. 7b), in agreement with the fact that ADPs and <RMSD^2^>^1/2^ of bound ligands increase only moderately in this series. In the latter case, differences in the configurational entropy contributions with respect to the length of the alkyl chain are close to zero (Fig. 7d). This is in agreement with the fact that ADPs and <RMSD^2^>^1/2^ of bound ligands increase more strongly in this series, thereby apparently keeping the loss of configurational entropy upon binding constant and small.

This view is corroborated if cAMP derivatives of the same chain length but with a terminal charge or N-acetylation are compared (Fig. 7e,f): the latter make favorable electrostatic and van der Waals interactions compared to the first. Configurational entropy contributions favor the short-chain cAMP derivative with a terminal charge, likely because its tail motion is less restricted due to the charge-charge repulsion in contrast to the charge-assisted hydrogen bonds formed by the N-acetyl group; for the longer tails, the differences in the configurational entropy contributions favor cAMP derivatives with N-acetylation, in agreement with the respective larger ADPs and <RMSD^2^>^1/2^ found for those derivatives. Taken together, an intricate interplay of favorable and disfavorable electrostatic interactions as well as differential configurational entropy losses can explain affinity gains of cAMP derivatives with longer alkyl chains and/or N-acetylation.

## Discussion

Herein we synthesized 20 mostly new cNMP derivatives modified at the 8-position of the purine moiety and studied the effects of these compounds on the activation gating of homotetrameric CNGA2, heterotetrameric CNGA2:CNGA4:CNGB1b and homotetrameric HCN2 channels. Notably, almost all of these cNMP derivatives are full agonists with potency at the best more than thirty times higher than the respective natural ligands cGMP (**1**_G_) or cAMP (**1**_A_). We show that alkyl chains of increasing lengths substantially enhance the apparent affinity of both CNGA2 and CNGA2:CNGA4:CNGB1b channels whereas their effects are much weaker in HCN2 channels, which have a high apparent affinity already for **1**_A_. Kinetic analyses of activation and deactivation time courses revealed that the effect of the alkyl chain is caused by a markedly slowed deactivation rate, suggesting that the dwell time of the cNMP derivatives at the CNBD is significantly prolonged when a hydrophobic residue is present in 8-position. Molecular docking and simulations in connection with end-point free energy calculations suggest that the measured differences in potency of the cAMP derivatives predominantly result from affinity differences.

In a previous report it has been shown that **1**_A_ and **1**_G_ bind in a different conformation to the CNBD: cAMP in *anti* and cGMP in *syn*^19^. For TAX-4 channels the *syn* conformation of cGMP has been verified recently^13^. The observed similarity of the effectivity pattern with cGMP and cAMP derivatives may support an earlier result^28^, suggesting that 8-substitution at the purine ring forces also cAMP derivatives to the *syn* conformation. This was, however, not confirmed by the present MD simulations of cAMP derivatives that were started from the *anti* conformation and displayed only moderate structural deviations of the cAMP head group, indicating the absence of steric strain for the 8-substituted adenine ring. Notably, for several analogs intermediate to cAMP and cGMP a consistent binding to isolated CNBDs of HCN2 in the anti conformation was shown by X-ray crystallography^45^. Though these cyclic nucleotides were not substituted as herein, this further supports the idea that the steric strains are only moderate.

Our most potent cNMP derivatives were those that contained a hexyl or decyl chain in 8-position of the purine ring. The *EC*_50_ values were 50, 140 and 15 nM for functional homotetrameric CNGA2, heterotetrameric CNGA2:CNGA4:CNGB1b and homotetrameric HCN2 channels, respectively (Figs 2 and 5c). To the best of our knowledge these apparent affinities are the highest for CNGA2 and HCN2 channels. For CNGA2:CNGA4:CNGB1b channels an even twofold lower *EC*_50_ value of 73 nM has been reported for 8-pCPT-cGMP^25^.

In a recent study the binding affinity of a large number of cAMP derivatives on isolated monomeric CNBDs of HCN1, HCN2 and HCN4 channels has been analyzed^29^ yielding for cAMP *BC*_50_ values between 2.0 and 3.7 µM. These values are much higher than typical *EC*_50_ values in functional channels in the order of 100 nM and below^36^. This suggests that isolated CNBDs alone are not able to generate these high affinities typical for functional channels with interacting subunits. This result also fits to previous results obtained by isothermal titration calorimetry^46^ showing that the cAMP binding to an isolated tetrameric CNBD of HCN2 has a high and a low affinity component and that in the monomeric CNBD the high affinity component is lost. A related result has also been reported recently for a single functional subunit in concatameric CNGA2 channels with only one functional CNBD: The *EC*_50_ value of its activating effect was approximately 22 times larger than that of the channel with four functional subunits^47^. When considering in the report of Moller and coworkers the effects of the best 8-substituted cAMP derivatives, 8-[Fluo]-cAMP and 8-Br-cAMP, on isolated monomeric CNBDs, the *BC*_50_ values ranged between 100 and 300 nM^29^. It remains to be tested whether these compounds generate significantly lower *EC*_50_ values in functional channels than in isolated CNBDs or whether these *BC*_50_ values for monomers are a lower limit. The latter idea is supported by the result that the *EC*_50_ value for cAMP binding to HCN2 channels with only one functional CNBD is similar to HCN2 channels with four functional CNBDs^48^, suggesting that, in contrast to CNGA2 channels, the subunits in HCN2 channels form high affinity CNBDs independent of the liganding of the other subunits.

The end-point free energy calculations yielded a very good correlation between computed binding free energies and experimentally determined p*EC*_50_ values, which is in contrast to numerous earlier results^42,49,50^. One reason may be the use of a high number of statistically independent conformations (4,000 snapshots extracted every 200 ps), resulting in precise (SEM < 0.7 kcal mol^−1^) computed binding free energies. Furthermore, here, the influence of charge differences in the cAMP derivatives may be less relevant than in previous studies^42^ because the tails remain largely accessible to the solvent. This view is also suggested by very similar trends in computed binding free energies irrespective of using *ε* = 1 or 4. For HCN2 channels, it has been observed that several conformational states in CNBDs regulate the binding of cNMPs^51^. It is therefore attractive to speculate that a similar mechanism regulates cNMP binding to CNG channels. Since our experimental results indicate that hydrophobic substituents in 8-position of cNMPs decrease the unbinding rate, we only considered the cNMP “trapped” state in our simulations, which should be the state that predominantly determines the affinities of the tested cNMP derivatives. A detailed investigation of energetic contributions with respect to tail length and presence or absence of N-acetylation revealed an intricate interplay of favorable and disfavorable electrostatic interactions as well as differential configurational entropy losses as underlying reasons for affinity differences. Notably, an increase in residual mobility of the tails of bound, N-acetylated cAMP derivatives results in configurational entropy contributions that become less unfavorable with increasing alkyl chain length compared to non-acetylated derivatives, while at the same time favorable electrostatic (and van der Waals) contributions decrease. This compensation is reflected in moderate decreases in *EC*_50_ values of these compounds by at most a factor of 15 (excluding **2**_**A**_) (Fig. 3b) despite adding four methylene units on going from 8-AET to 8-AHT and 8-AHT to 8-ADT; for comparison, for a single buried methyl group, increases of association constants at 298 K by a factor of 3–11 have been estimated^52–55^. The cAMP derivatives investigated here computationally thus reflect an example of enthalpy/entropy compensation similarly observed for *para*-substituted benzenesulfonamides with “greasy tails” that bind to bovine carbonic anhydrase II, for which a model of decreasing “tightness” of the protein−ligand interface as the chain length of the ligand increases was proposed^56^. Note that in our context “enthalpy” relates to changes in electrostatic and van der Waals interactions and “entropy” to changes in the configurational entropy, as the polar and non-polar parts of solvation free energies contain both enthalpic and entropic components (see Homeyer and coworkers^57^ for further discussion). A sequence alignment of human, murine and rat HCN1/2 and CNGA1/2 (Supplementary Fig. 5) revealed a high similarity between the residues of rCNGA2 that contact the investigated cNMP derivatives and the corresponding residues in HCN channels, which suggests a similar mechanism of enthalpy - entropy compensation in HCN channels. This may provide an explanation for the highly similar *EC*_50_ values of the ligands in CNG and HCN channels, respectively (Fig. 5c). This sequence alignment also reveals distinct (R632) and homologous (R635, which corresponds to K582 in rCNGA2) residues in HCN channels that are suggested to be involved in potential charge-charge repulsions between *N*-terminally charged C_2_ derivatives and CNG channels. It is of note that, to the best of our knowledge, the other residues involved in these charge-charge repulsions in CNG channels (Fig. 6b), have not yet been characterized in other studies^19,58,59^.

It should also be noted that the structure of the CNBDs in CNG and HCN channels is similar to that of other proteins with entirely different function including protein kinase A, exchange protein directly activated by cAMP (Epac) and the prokaryotic cAMP receptor CAP^60,61^. The interactions of cyclic nucleotides with the CNBDs of these proteins have been intensively studied^62–66^. It would be of great interest to test the effectivity of the highly affine cNMP derivatives described herein also on these proteins.

Together, our results provide a strategy to generate cNMPs with higher affinity to CNG and HCN channels. On the one hand, this strategy has potential for studying the process of ligand binding, in particular at the single-channel level, because the dwell time of these cNMP derivatives is presumably considerably prolonged. Hence, an appropriate coupling of dyes to these cNMP derivatives is promising to study in single channels individual steps of ligand binding and activation gating in parallel, analogous to previous studies in macropatches^34,36^. For one fluorescent cAMP derivative, containing the hexyl chain studied herein, its usefulness has been demonstrated: In combination with mutagenesis strategies the binding to defined subunits in CNGA2:CNGA4:CNGB1b channels could be quantified^67^. Furthermore, our high-affinity cNMP derivatives should also be of interest for the development of specific drugs targeting HCN or CNG channels to treat such severe diseases as bradycardia^68^ or achromatopsia^69^.

## Methods

### Synthesis of cNMP derivatives

The cyclic nucleotides cGMP and cAMP were obtained from Sigma. All synthesized derivatives were monitored and analyzed by reversed phase HPLC and thin layer chromatography (TLC), visualization was effected by UV (254 nm) and confirmed by mass spectroscopy. The structures of all C8-modified cNMP derivatives are given in Table 1. Three of the analogs (**2**_G_, **4**_A/G_) were previously studied in other channel types or isolated CNBDs^29,30^.

8-Br-cAMP and 8-Br-cGMP (Biolog Life Science Institute, Bremen) are appropriate compounds for the synthesis of the C8 substituted cAMP and cGMP derivatives, respectively. Following the synthesis route described by Brown *et al*.^30^ with minor modifications, the 8-Br-cNMP was first substituted to the thiol by thiourea. The desired thioether was then obtained with an appropriate linker derivative. The respective linker derivative comprised an N-terminal protected bromoalkylamine or bromoheteroalkylamine. The compounds described herein were obtained by deprotection of the terminal amino group and part of them was further acetylated. A detailed description of the synthesis is provided in Supplementary Methods.

### Molecular Biology and functional expression

The subunits CNGA2 (accession No. AF126808), CNGA4 (accession No. U12623) and CNGB1b (accession No. AF068572) of rat olfactory channels as well as mouse HCN2 channels (NM008226) were subcloned in front of the T7 promoter of pGEMHEnew. The respective cRNAs were produced by using the mMESSAGE mMACHINE T7 Kit (Ambion, Austin, TX).

Oocytes of *Xenopus laevis* were obtained either from Ecocyte® (Castrop-Rauxel, Germany) or surgically under anesthesia (0.3% 3-aminobenzoic acid ethyl ester) from female adults. The procedures had approval from the authorized animal ethical committee of the Friedrich Schiller University Jena. The methods were carried out in accordance with the approved guidelines.

The oocytes were exposed to collagenase A (3 mg/ml; Roche, Grenzach-Wyhlen, Germany) for 105 min in Ca^2+^-free Barth’s solution containing (in mM) 82.5 NaCl, 2 KCl, 1 MgCl_2_ 2, and 5 Hepes, pH 7.4. Oocytes at stages IV and V were injected with 50–130 ng of cRNA encoding either CNGA2, CNGA2:CNGA4:CNGB1b (2:1:1 ratio) or HCN2 channels either manually of or by means of an injection robot (RoboInject®). The injected oocytes were incubated at 18 °C for up to 6 days in Barth’s solution containing (in mM) 84 NaCl, 1 KCl, 2.4 NaHCO_3_, 0.82 MgSO_4_, 0.41 CaCl_2_, 0.33 Ca(NO_3_)_2_, 7.5 TRIS, cefuroxime (4.0 µg × ml^−1^), and penicillin/streptomycin (100 µg × ml^−1^), pH 7.4.

### Electrophysiology

Macroscopic currents were recorded from inside-out patches of the oocytes by using standard patch-clamp techniques. The patch pipettes were pulled from quartz tubing (P-2000, Sutter Instrument, Novato, USA) with an outer and inner diameter of 1.0 and 0.7 mm (VITROCOM, New Jersey, USA). The corresponding pipette resistance was 0.9–2.3 MΩ. The bath and pipette solution contained (in mM): 150 KCl, 1 EGTA, 5 Hepes (pH 7.4 with KOH) for CNG measurements. For HCN measurements the bath solution contained 100 mM KCl, 10 mM EGTA, 10 mM HEPES (pH 7.2) and 120 mM KCl, 10 mM HEPES, and 1.0 mM CaCl2 (pH 7.2) in the pipette. Recording was carried out at room temperature using an Axopatch 200B amplifier (Axon Instruments, Foster City, CA). Electrophysiology was controlled by the Patchmaster-software (HEKA Elektronik Dr. Schulze GmbH, Lambrecht, Germany). The sampling rate was 5 kHz and the filter implemented in the amplifier (4-pole Bessel) was set to 2 kHz. Measurements in HCN2 channels were generally started 3.5 min after patch excision to minimize channel run down during the measurement^14,27,70^. The solutions with the ligand concentrations to be tested were applied via a multi-barrel device to the patches with a flow rate of 0.8 to 1.2 ml/min.

In experiments designed to record activation and deactivation kinetics in CNG channels, theta-glass pipettes were used in which one barrel contained control solution and the other barrel the test solution supplemented with the ligand. The theta-glass pipette was mounted on a piezo actuator which was controlled by the computer controlling the voltage protocols. The effective switch time of the solution exchange, *t*_10–90_, determined by an open patch pipette and different solutions in the barrels^71^, was negligible compared to the time courses of activation and deactivation to be considered. The concentration of all ligands was verified by UV spectroscopy.

### Fitting steady-state concentration-activation relationships by Hill functions

Concentration-activation relationships were fitted with the Igor software® by

1 $$l/{l}_{{\rm{\max }}}=1/(1+E{C}_{50}/{[{\rm{CN}}]}^{H}).$$

*I* is the actual current amplitude and *I*_max_ the maximum current amplitude at a saturating cNMP concentration specified for each cNMP and channel. *EC*_50_ is the cNMP concentration generating the half maximum current and *H* the Hill coefficient.

The speed of activation and deactivation was determined by fitting the respective time courses with single exponentials yielding the time constant *τ*. Part of the time courses required the sum of two exponentials for adequate description, yielding the time constants *τ*_1_ and *τ*_2_ and the contributions A_1_ and A_2_, respectively. From these a weighted mean time constant was computed according to

2 $${\tau }_{{\rm{wm}}}=({{\rm{A}}}_{1}{\tau }_{1}+{{\rm{A}}}_{2}{\tau }_{2})/({{\rm{A}}}_{1}+{{\rm{A}}}_{2}).$$

To quantify the time course at a given cNMP concentration for a number of measurements τ_wm_ and τ were treated equally. Errors are given as mean ± s.e.m. Statistical tests were performed by the Igor software®. The *t*-test level of significance was set to 0.01 with respect to cGMP or cAMP.

### Molecular modelling and simulations

In order to explain the observed structure/apparent affinity relationships of the non-acetylated and N-acetylated cAMP derivatives **2**_A_–**7**_A_, we first generated a homology model of homotetrameric rat CNGA2 using the cryo-EM structure of the cyclic nucleotide-gated cation channel TAX-4 from *C. elegans* in the cGMP-bound open state (PDB-ID: 5H3O)^13^ as a template. Molecular docking was then used to generate complex structures of derivatives of the congeneric series 8-AET-cAMP (**2**_A_), 8-(Ac)AET-cAMP (**3**_A_), 8-AHT-cAMP (**4**_A_), 8-(Ac)AHT-cAMP (**5**_A_), 8-ADT-cAMP (**6**_A_), and 8-(Ac)ADT-cAMP (**7**_A_) and the homology model of CNGA2. The resulting six complex structures were subjected to molecular dynamics (MD) simulations in conjunction with binding free energy calculations. Details are given in Supplementary Methods.

## Electronic supplementary material

Supplementary Information
