# Supplementary material for: Hydrophobic alkyl chains substituted to the 8-position of cyclic nucleotides enhance activation of CNG and HCN channels by an intricate enthalpy - entropy compensation
Source: Sci Rep. 2018 Oct 8;8:14960. doi: 10.1038/s41598-018-33050-5 (PMC6175941; doi:10.1038/s41598-018-33050-5)
Supplement: Supplementary file 1 — Supplementary Information [file 41598_2018_33050_MOESM1_ESM.docx]

## Supplementary Information

to

**Hydrophobic alkyl chains substituted to the 8‑position of cyclic nucleotides enhance activation of CNG and HCN channels by an intricate enthalpy ‑ entropy compensation**

by Maik Otte, Andrea Schweinitz, Michele Bonus, Uta Enke, Christina Schumann, Holger Gohlke, Klaus Benndorf

## Supplementary Methods

### Chemical Syntheses

#### General

All reagents were of analytical or HPLC grade and were purchased from commercial suppliers.

Reactions were followed by analytical reversed phase HPLC at 30°C with a JASCO PU-2080 Plus system (JASCO, Labor- und Datentechnik GmbH, Gross-Umstadt, Germany) consisting of a DG‑2080-53 In‑Line Degasser, a PU-2080 pump, a column oven (CO-2060 Plus) and a UV-2075 detector using a linear water (A)-methanol (B) gradient (increase of 1 % B/min) both containing 0.1 % formic acid at a flow rate of 1 ml/min. A 250 x 4.6 mm column filled with OTU LipoMare C_18_ (5 µm, 105 Å) as stationary phase including an OTU LipoMare C18 5 µm (10 x 4.6 mm, 105 Å) precolumn cartridge (all AppliChrom, Oranienburg, Germany) were used.

Semipreparative HPLC purification was performed on a JASCO chromatograph PU-2087 Plus system equipped with a DG-2080-54 In-Line Degasser, a PU-2087 pump and a UV-975 UV detector using an OTU LipoMare C_18_ column (5 µm 250 x 8 mm, flow rate 2 ml/min) including a 30 x 8 mm precolumn. Elution was achieved running a linear water (A)-methanol (B) gradient (increase of 45 % B in 120 min) with 0.1 % formic acid, in some cases with 5 mM ammonium acetate (pH 5) was used. Column effluents were monitored at 254 nm. Both HPLC systems were managed by ChromPass Chromatography DataSystem software version 1.8.6.1.

MS characterization was performed by High-resolution UHPLC-MS on an Accela UHPLC system connected to a Q Exactive Orbitrap (both Thermo Scientific). The system was operated in the ESI-MS mode and full scan mass spectra (m/z 100 to 1550) were measured. The UHPLC was run on an RP C18 column (Accucore C_18_ 100 x 2.1 mm), developed by a flow rate of 0.2 ml/min with a water-acetonitrile gradient containing 0.1 % formic acid as mobile phases. A linear gradient was used starting from 5 % to 98 % acetonitrile in 14 min. Data acquisition and processing was controlled by Thermo Scientific Xcalibur software.

Thin layer chromatography (TLC) was performed on silica gel plates (silica gel 60 F254, Merck, Darmstadt, Germany) in n-butanol/acetic acid/water 5/3/2 (v/v/v) visualization was effected by UV (250 nm).

### Synthesis of Precursor 8-thio-cGMP and 8-thio-cAMP

The starting material 8-bromo-cGMP or 8-bromo-cAMP (sodium salt) was purchased from Biolog Life Science Institute (Bremen, Germany) and was converted into the corresponding thiol in accordance with the reported procedure by Brown et al.^1^ with minor changes.

Briefly, 50 mg (0.12 mmol) of 8-bromo-cGMP (sodium salt) and a tenfold excess of thiourea (85 mg, 1.2 mmol) were dissolved in 1 ml DMSO and heated on a heating block at 110 °C. The progress of the reactions was monitored by analytical reversed phase HPLC. After completion of the reactions the volume of the raw mixtures was reduced to ~100 μL in a vacuum concentrator RVC 2-18 CDplus (Christ, Osterode, Germany). The crude product was precipitated first in acetone to remove excess of thiourea and then twice with tert-Butyl methyl ether (TBME) to give a pale yellow solid with > 95 % purity (area % HPLC) based on UV absorbance to be used without further purification. Successful synthesis was confirmed by mass spectrometry (8-thio-cGMP: HPLC R_t_ = 13.9 min; TLC R_f_ = 0.55; MS calc. 377.02, MS found m/z: 376.01 (M-H)^-^, 400.0 (M+Na)^+^). The same procedure was used in parallel for 8-thio-cAMP (8-thio-cAMP: HPLC R_t_ = 12.5 min; TLC R_f_ = 0.65; MS calc. 361.02, MS found m/z: 360.02 (M-H)^-^, 362.03 (M+H)^+^).

### Synthesis of 8-AHT-cGMP (4_G_) and 8-(Ac)AHT-cGMP (5_G_)

8-thio-cGMP (10 mg, 25 µM) and N-(6-Bromohexyl)-phthalimide (16 mg, 50 µM, Alfa Aesar, Karlsruhe, Germany) were dissolved in 1 ml methanol and brought to pH ~ 10 by addition of sodium methoxide in accordance to Caretta et. al.^2^. The mixture was held on 40 °C for 2 h. When the HPLC analysis indicated that the reaction was complete, the mixture was centrifuged the resulting supernatant was concentrated and precipitated in TBME. The N-terminal phthaloyl group was then cleaved with hydrazine hydrate in ethanol containing 5 % water at 80 °C, followed by 6M HCl^3^. The progress of the reaction was monitored by analytical HPLC. Finally, the mixture was adjusted to neutral pH with NaOH, centrifuged and the resulting deposit precipitated with TBME. The crude product was purified by semipreparative HPLC, followed by lyophilization. The overall yield after purification was 42 % with a purity of > 99 % (HPLC area %) based on UV absorbance (8-AHT-cGMP (**4**_G_): HPLC R_t_ = 21.9 min; TLC R_f_ = 0.34; MS calc. 476.12, MS found m/z: 475.12 (M-H)^-^, 499.11 (M+Na)^+^). Part of the product **4**_G_ (3 mg, 6 µM) was acetylated with a 5 fold excess of acetic anhydride in water/MeOH 2/1 (v/v). The reaction was maintained at pH ~ 8 by the addition of NH_4_OH (30 %). The progress was monitored by analytical HPLC. After completion the solvent was removed under reduced pressure. The residue was purified by semipreparative HPLC. After lyophilisation the product was obtained as white powder. Successful synthesis was confirmed by mass spectrometry (8‑(Ac)AHT-cGMP **(5**_G_**)**: HPLC R_t_ = 32,1 min; TLC R_f_ = 0.55; MS calc. 518.13, MS found m/z: 517.13 (M-H)^-^).

The other compounds used herein were synthesized in an analogue manner. Successful synthesis for all compounds was confirmed by mass spectrometry. Analytical details are summarized in Supplementary Table 3.

### Synthesis of 8-AET-cGMP (2_G_) and 8-AET-cAMP (2_A_)

For **2**_G_ and **2**_A_ the alkylation was done without protecting group und further deprotection. Therefore, 8‑thio-cGMP and 8-thio-cAMP were alkylated with 2-Bromoethan-1-amine hydrobromide (Sigma, Darmstadt, Germany), respectively. The compounds were obtained as white powder after lyophilisation. Successful synthesis was confirmed by mass spectrometry. For analytical details see Supplementary Table 3.

### Synthesis of 8-AHT-cAMP (4_A_), 8-ADT-cGMP (6_G_) and 8-ADT-cAMP (6_A_)

N-(6-Bromohexyl)-phthalimide (Alfa Aesar, Karlsruhe, Germany) for compound **4**_A_ and N‑(10‑Bromodecyl)-phthalimide (abcr, Karlsruhe, Germany) for compounds **6**_G_ and **6**_A_ were added to the corresponding 8-thio-cAMP or 8-thio-cGMP as described for **4_G_** The compounds were obtained as white powder after lyophilisation. Successful synthesis was confirmed by mass spectrometry. For analytical details see Supplementary Table 3.

### Synthesis of 8-(Ac)AET-cGMP (3_G_), 8-(Ac)ADT-cGMP (7_G_), 8-(Ac)AET-cAMP (3_A_), 8-(Ac)AHT-cAMP (5_A_), 8-(Ac)ADT-cAMP (7_A_)

Acetylation was conducted similarly to the synthesis of **5**_G_ using **2**_G_, **6**_G_, **2**_A_, **4**_A_, and **6**_A_ for the respective synthesis of **3**_G_, **7**_G_, **3**_A_, **5**_A_, and **7**_A_. The compounds were obtained as white powder after lyophilisation. Successful synthesis was confirmed by mass spectrometry. For analytical details see Supplementary Table 3.

### Synthesis of 8-(Ac)APT-cGMP (8_G_), 8-(Ac)APT-cAMP (8_A_)

BOC-Amino-(PEG)_2_-Br (BIOZOL, Eching, Germany) was coupled to the corresponding 8-thio-cGMP or 8-thio-cAMP for the respective synthesis of **8**_G_ and **8**_A_, as described for the synthesis of **4**_G_. Deprotection was done with 1M HCl in acetic acid using a standard procedure. Further acetylation was conducted similarly to the synthesis of **5**_G_. The compounds were obtained as white powder after lyophilisation. Successful synthesis was confirmed by mass spectrometry. For analytical details see Supplementary Table 3.

### Synthesis of 8-(Ac)APET-cGMP (9_G_), 8‑(Ac)APET‑cAMP (9_A_) and 8-(Ac)AGET-cGMP (10_G_), 8‑(Ac)AGGET-cGMP (11_G_), 8‑(Ac)AGET‑cAMP (10_A_), 8‑(Ac)AGGET-cAMP (11_A_)

**2**_G_ and **2**_A_ were coupled to Fmoc-Adoa-OH (Iris Biotech, Marktredwitz, Germany) using a standard PyBOP coupling method for the respective synthesis of **9**_G_ and **9**_A_ and to Boc-Gly-OSu (Aldrich, Darmstadt, Germany) for the respective synthesis of **10**_G_, **10**_A_ . Additionally, Boc-Gly-OSu was coupled to **10**_G_ and **10**_A_ for the synthesis of **11**_G_, **11**_A_.

Deprotection was done with 20 % piperidin in dimethylformamide for the Fmoc- and 1M HCl in acetic acid for the Boc-protecting group using common procedures. Further acetylation was conducted similarly to the synthesis of **5**_G_. The compounds were obtained as white powder after lyophilisation. Successful synthesis was confirmed by mass spectrometry. For analytical details see Supplementary Table 3.

### Homology modeling of the homotetrameric CNGA2 channel structure

A homology model of homotetrameric rat CNGA2 (UniProt accession ID: Q00195) was generated using the 3.5 Å cryo-EM structure of the cyclic nucleotide-gated cation channel TAX-4 from *C. elegans* in the cGMP-bound open state (PDB ID: 5H3O)^4^ as a template. Target-template alignment (sequence identity: ~53 %, coverage: ~77 %, target residues: 133-583 of each monomer) and structural modeling were carried out using the SWISS-MODEL workspace^5^. The C_4_ symmetry of the template structure was preserved in the final model. Model quality was assessed with the server implementation of the MolProbity software^6^, version 4.4 and the visualization software KiNG^7^, version 2.23: a Clashscore of 1.72 and a MolProbity score of 1.55 (99^th^ and 94^th^ percentile, respectively) indicated a well-defined structure. No clashes or geometry outliers were detected in or near the cyclic nucleotide-binding domains.

### Parameterization of cAMP derivatives

To derive a consistent set of point charges and force field parameters for the cAMP derivatives, the ligand set was split into a core fragment and ten side chain fragments (Supplementary Fig. 1). In particular, 8-methylthio-cAMP was involved in the charge derivation for the core fragment, and the respective *S*-substituted 1-methyl-1*H*-imidazole-2-thiol derivatives were involved in the charge derivation for the side chain fragments.

#### Construction of molecular fragments for charge derivation

To generate three-dimensional structures of 8-methylthio-cAMP, the structure of cAMP was obtained from the crystal structure of the hyperpolarized-activated cyclic nucleotide-gated ion channel 2 (HCN2, PDB ID: 1Q5O)^8^. A methylthio substituent was added to the nucleotide using the 3D builder of the Maestro (Schrödinger, LLC)^9^ GUI, and free valences were filled with hydrogens while preserving the negative charge at the cyclic phosphate. Three-dimensional structures of the *S*‑substituted 1-methyl-1*H*-imidazole-2-thiol derivatives were prepared accordingly. To prevent the formation of intramolecular interactions in the following geometry optimization step, the alkyl chains in these fragments were constructed to adopt an all-anti conformation.

#### Derivation of atom-centered point charges for the molecular fragments

To obtain charges for the molecular fragments, a gas-phase geometry optimization of each fragment was carried out at the HF/6-31G(d) level using GAUSSIAN 09, Revision B.01^10^. To ensure invariance of the molecular electrostatic potential (MEP) with respect to molecular orientation, the MEP computation (level of theory: HF/6-31G(d)) and charge fitting procedures were carried out on the R.E.D. server^11^, which uses a rigid-body reorientation algorithm^12^ before computing the electrostatic potential. The RESP procedure^13^ with two fitting stages (hyperbolic constraint values: 0.0005/0.001) was used for charge derivation^14,15^. The MEP was calculated on four layers defined by scaling the atomic van der Waals radii by a factor of 1.4, 1.8, 2.0 and 2.2, respectively, and a point density of 0.28 points au^-2^ (1 pt Å^-2^). During charge fitting, additional inter-molecular charge constraints between the methyl group of the 8-methylthio-cAMP and the (1-methyl-1*H*-imidazolyl-2-yl)sulfanyl portion of the *S*-substituted 1-methyl-1*H*-imidazole-2-thiol derivatives with a target value of zero were employed.

#### Derivation of atom types and missing force field parameters for the cAMP derivatives

Three-dimensional structures of the cAMP derivatives were constructed from the molecular fragments in the xLEaP module of AmberTools17^16^. Atom types from the GAFF force field^17^ were assigned to each structure using the antechamber program (AmberTools17), and missing force field parameters were determined using the parmchk module^18^ (AmberTools17), respectively. The missing dihedral parameters for the na‑cc‑ss‑c3 and the nd‑cc‑ss‑c3 torsions were derived from ab initio calculations. In detail, using 2-(methylthio)-1*H*-imidazole as a model compound, a quantum-mechanically (QM)-derived energy profile of the 360° rotation about the C-S bond in the gas phase was computed in increments of 8° at the HF/6-31G(d) level using GAUSSIAN 09, Revision B.01^10^. For each of the 46 output structures, the molecular mechanics (MM) energy was evaluated using the GAFF force field^17^, while setting the values of the missing torsion parameters to zero. The values for the potential energy function that need to be described by the missing dihedral parameters to match the QM energies were then calculated as the difference between the QM-derived energy profile and the MM-derived energy profile. The missing parameters were derived using a global optimization strategy.

### Generation of cAMP derivatives/CNGA2 complex structures

Structures for the protein-ligand complexes of the cAMP derivatives and the CNGA2 tetramer were generated by molecular docking using the Maestro (Schrödinger, LLC)^9^ GUI and the Glide XP methodology^19^. The structure of the CNGA2 homology model was prepared for docking using the default settings of the Protein Preparation Wizard built into Maestro (Schrödinger, LLC)^9^. Protein protonation states were determined using the built-in implementation of PROPKA^20,21^. No minimization was performed to preserve C_4_ symmetry. To determine the optimal location of the docking grid in a CNGA2 monomer, chain A of the cGMP-bound TAX-4 cryo-EM structure^4^ was superimposed onto chain A of the CNGA2 homology model. After this superimposition, the coordinates of the center of mass of cGMP in the TAX-4 structure were used as coordinates for the center of the docking grid in CNGA2. The volume of the inner box of the docking grid was set to 15 Å^3^, and the dimensions of the outer box were set such that ligands with a length of ~20 Å could be docked. No constraints, rotatable groups, or excluded volumes were defined. Docking was performed using the default settings of the Glide XP methodology as implemented in the Schrödinger Suite v. 2017-1. After completion of the docking runs in chain A, the coordinates of the ligands were duplicated into the other three chains.

### Molecular dynamics simulations of cAMP/CNGA2 complexes

#### System setup

The orientation of the CNGA2 tetramer in the membrane was predicted using the PPM web server^22^. The calculated transfer free energy of 112.1 kcal/mol and the predicted tilt angle of 0.0 ± 0.0° indicated a high reliability of the predicted orientation. This orientation was then used to embed the simulation systems into a lipid bilayer, consisting of ~80 % DOPC (upper leaflet: 168, lower leaflet: 206) and ~20 % POPC (upper leaflet: 44, lower leaflet: 40) molecules in both membrane leaflets using CHARMM-GUI^23^. The minimum water height on the top and bottom of the system was chosen to be 25 Å. The program was then invoked to add 154 mM sodium chloride. The final system comprised ~264,000 atoms. To convert the pdb file created with CHARMM-GUI^23^ into a pdb file recognized by Amber and the AmberTools programs, the charmmlipid2amber.py script of AmberTools17 was used. Topology files were built using the tLEaP module in AmberTools17. The ff14SB force field^24^ was used for the protein, the Lipid14 force field^25^ was used for the lipids, and the GAFF force field^17^ with the modified parameters was used for the ligands.

#### Equilibration protocol

All MD simulations were carried out using the mixed precision SPFP GPU implementation of the Amber 17 package of molecular simulation programs^16,26^. A time step of 2 fs was used for integration, and the Langevin thermostat^27^ was used for temperature control with a collision frequency of γ = 0.01 ps^-1^ and a target temperature of *T =* 300 K. Covalent bonds involving hydrogen atoms were constrained using the SHAKE algorithm^28^. The Particle Mesh Ewald^29^ method was used to estimate long-range electrostatic interactions, and a cutoff of 10 Å was used for short-range electrostatics and van der Waals forces.

The initial structures were energy minimized for 5,000 steps using the steepest descent algorithm, followed by 5,000 steps of minimization with the conjugate gradient algorithm; during this procedure, all protein and membrane atoms were restrained to their initial positions by harmonic restraints with a force constant of 25.0 kcal mol^-1^ Å^-2^. This step was first repeated with the force constant of the harmonic restraints lowered to 5.0 kcal mol^-1^ Å^-2^, then repeated with the restraints removed from the lipid atoms, and, finally, repeated without restraints. After the final minimization step, the root-mean-square deviation (RMSD) of the atomic positions of the backbone atoms was ≤ 0.73 Å for all systems with respect to the initial structure. 5 ps of NVT-MD (protein restrained; force constant: 5.0 kcal mol^-1^ Å^-2^) were performed while heating the system from 0 K to 100 K. 115 ps of NPT-MD (protein restrained; force constant: 5.0 kcal mol^-1^ Å^-2^) were performed for density adaptation while heating the system from 100 K to 300 K. After removing the restraints, additional 4,880 ps of NPT-MD were performed.

#### Production protocol

For each of the 6 complex structures, 200 ns of NPT-MD were carried out, resulting in an aggregate simulation time of 1.2 µs. Coordinates for analysis and post-processing were saved every 20 ps.

Post-processing and data analysis

Post-processing and analysis of the MD trajectories was performed in CPPTRAJ^30^ as implemented in AmberTools17.

### Binding free energy calculations

All calculations described in the following subsections were performed using the MD trajectories of those CNGA2 complexes that contain ligands of the congeneric series 8-AET-, 8-(Ac)AET-, 8-AHT-, 8-(Ac)AHT-, 8-ADT-, and 8-(Ac)ADT-cAMP. For 8-(Ac)APT-, 8-(Ac)APET-, 8-(Ac)AGET-, and 8‑(Ac)AGGET-cAMP, no such calculations were performed, because their p*EC*_50_ values span < 0.3 log units.

Calculation of effective binding energies

For each simulation system, the change in effective energy due to ligand binding ($\Delta G_{\text{eff}}^{\text{MM-PBSA}}$) was calculated by the single-trajectory MM-PBSA approach^31-33^ using the MMPBSA.py module^34^ as implemented in AmberTools17^16^. In short, for a set of simulation snapshots $i=\left[1,N\right]$, where *N* is the total number of snapshots selected for analysis, $\Delta G_{\text{eff, }i}^{\text{MM-PBSA}}$ was calculated as the sum of the contributions to the molecular mechanics free energy difference ($\Delta G_{i}^{\text{MM}}$) and the solvation free energy difference ($\Delta G_{i}^{\text{solv}}$) (eq. 1):

$\Delta G_{\text{eff, }i}^{\text{MM-PBSA}}=\Delta G_{\text{elec, }i}^{\text{MM}}+\Delta G_{\text{vdW, }i}^{\text{MM}}+\Delta G_{\text{int, }i}^{\text{MM}}+\Delta G_{\text{nonpol, }i}^{\text{solv}}+\Delta G_{\text{pol, }i}^{\text{solv}}$ (eq. 1)

where $\Delta G_{\text{elec}}^{\text{MM}}$, $\Delta G_{\text{vdW}}^{\text{MM}}$, and $\Delta G_{\mathrm{int}}^{\text{MM}}$ are contributions of the electrostatic, van der Waals, and internal energies, and $\Delta G_{\text{nonpol}}^{\text{solv}}$ and $\Delta G_{\text{pol}}^{\text{solv}}$ are the nonpolar and polar parts of the solvation free energy. The effective binding energy of each ligand to CNGA2 was then expressed as an average over all snapshots (eq. 2):

$\Delta G_{\text{eff}}^{\text{MM-PBSA}}=\frac{\sum_{i=1}^{N} \Delta G_{\text{eff, }i}^{\text{MM-PBSA}}}{N}$ (eq. 2)

with standard error of the mean (SEM; eq. 3)

$\mathrm{SEM}\left(\Delta G_{\text{eff}}^{\text{MM-PBSA}}\right)=\frac{s\left(\Delta G_{\text{eff}}^{\text{MM-PBSA}}\right)}{\sqrt{N}}$ (eq. 3)

where *s* denotes the sample standard deviation.

In this study, $\Delta G_{\text{pol}}^{\text{solv}}$ was calculated by the linear Poisson-Boltzmann (PB) equation using an ionic strength of 100 mM, internal dielectric constants ($\varepsilon_{\text{int}}$) of 1 and 4, and an external dielectric constant
($\varepsilon_{\text{ext}}$) of 80. Solutions for the linear PB equation were computed with 1,000 iterations on a cubic (lattice) grid with 0.5 Å spacing between grid points. $\Delta G_{\text{nonpol}}^{\text{solv}}$ was considered proportional to the solvent accessible surface area (SASA) according to eq. 4:

$\Delta G_{\text{nonpol}}^{\text{solv}}=\gamma\left(\text{SASA}\right)+\beta+E_{\text{dispersion}}$ (eq. 4)

with *γ* = 0.0378 kcal mol^-1^ Å^-2^ and *β* = -0.5692 kcal mol^-1^. The SASA was calculated with a solvent probe radius of 1.4 Å, Tan & Luo radii^35^ for the protein, and mbondi radii^36^ for the ligands. The dispersion term was calculated by a surface-integration approach^37^.

For each simulation, $\Delta G_{\text{eff}}^{\text{MM-PBSA}}$ was calculated from 1,000 simulation snapshots (time between snapshots: 200 ps). Since the simulated CNGA2 tetramer systems contained a ligand bound to each CNBD, the effective binding energies were calculated as averages over all CNBDs (eq. 5):

$\Delta G_{\text{eff}}^{\text{MM-PBSA}}=\frac{\Delta G_{\text{eff, CNBD1}}^{\text{MM-PBSA}}+\Delta G_{\text{eff, CNBD2}}^{\text{MM-PBSA}}+\Delta G_{\text{eff, CNBD3}}^{\text{MM-PBSA}}+\Delta G_{\text{eff, CNBD4}}^{\text{MM-PBSA}}}{4}$ (eq. 5)

with standard error of the mean (eq. 6):

$\mathrm{SEM}\left(\Delta G_{\text{eff}}^{\text{MM-PBSA}}\right)=\sqrt{\mathrm{SEM}\left(\Delta G_{\text{eff, CNBD1}}^{\text{MM-PBSA}}\right)^{2}+\mathrm{SEM}\left(\Delta G_{\text{eff, CNBD2}}^{\text{MM-PBSA}}\right)^{2}+\mathrm{SEM}\left(\Delta G_{\text{eff, CNBD3}}^{\text{MM-PBSA}}\right)^{2}+\mathrm{SEM}\left(\Delta G_{\text{eff, CNBD4}}^{\text{MM-PBSA}}\right)^{2}}$ (eq. 6)

#### Calculation of the entropic contribution to binding

Similarly, for each simulation system, the changes in configurational entropy due to ligand binding
($\Delta S_{\text{NM}}$) was calculated by normal mode analysis (NMA) as implemented in MMPBSA.py. In short, for a subset of simulation snapshots $i=\left[1,N\right]$, where *N* is the total number of snapshots selected for analysis, $\Delta S_{\text{NM, }i}$ was calculated as the sum of the contributions that arise from changes in translational ($\Delta S_{\text{trans, }i}$), rotational ($\Delta S_{\text{rot, }i}$), and vibrational ($\Delta S_{\text{vib, }i}$) entropy:

$\Delta S_{\text{NM, }i}=\Delta S_{\text{trans, }i}+\Delta S_{\text{rot, }i}+\Delta S_{\text{vib, }i}$ (eq. 7)

The change in configurational entropy after ligand binding was then estimated as the average over all *N* snapshots:

$\Delta S_{\text{NM}}=\frac{\sum_{i=1}^{M} \Delta S_{\text{NM, }i}}{N}$ (eq. 8)

with SEM:

$\mathrm{SEM}\left(\Delta S_{\text{NM}}\right)=\frac{s\left(\Delta S_{NM}\right)}{\sqrt{N}}$ (eq. 9)

A convergence criterion of 0.001 kcal mol^-1^ Å^-1^ for minimization and the HCT Generalized Born model for solvation^38^. Due to the computational expense, all calculations were performed on truncated systems that only contain the ligand and the first and second shell of the binding site residues. It has been suggested that such an approach gives rise to a large statistical uncertainty of the calculated entropies^39,40^, which probably results from larger structural changes in the truncated systems during minimization compared to the complete systems. Solutions to this problem, such as the consideration of a buffer region around the truncated systems that is kept fixed during minimization^41^, have been brought forward. However, in these studies, comparably short MD trajectories (~1-2 ns) were used for the MM-GBSA/PBSA calculations. Since we evaluate here 1,000 snapshots from a 200 ns trajectory for four binding sites, we do not expect the uncertainty in our estimate of the entropy term to become a limiting factor. This expectation is confirmed by a maximal SEM(Δ*S*_NM_) = 0.41 kcal mol^-1^ found for 8‑ADT-cAMP (data not shown).

#### Calculation of binding free energies

After effective binding energies and contributions from changes in configurational entropy were estimated for each complex, binding free energies ($\Delta G_{\text{bind}}$) were calculated according to (eq. 10):

$\Delta G_{\text{bind}}=\Delta G_{\text{eff}}^{\text{MM-PBSA}}-T\Delta S_{\text{NM}}$ (eq. 10)

with *T* = 298.15 K and SEM (eq. 11):

$\mathrm{SEM}\left(\Delta G_{\text{bind}}\right)=\sqrt{\text{SEM}\left(\Delta G_{\text{eff}}^{\text{MM-PBSA}}\right)^{2}+T^{2} \text{SEM}\left(\Delta S_{\text{NM}}\right)^{2}}$ (eq. 11)

## Supplementary Results

### Validation of force field parameters

To assess whether the newly derived na-cc-ss-c3 and nd-cc-ss-c3 dihedral parameters improve the agreement with QM-derived energies, the QM energies of 46 conformers of 2-(methylthio)-1*H*-imidazole, generated by rotation about the na-cc-ss-c3 torsion in steps of 8°, were compared with their respective MM energies obtained with either the parmchk2-derived parameters or the new parameters. While the dihedral parameters obtained from the parmchk2 tool underestimate the torsional energy profile by, on average, 0.89 kcal mol^-1^ (MAE), the newly derived parameters reduce this error to, on average, 0.09 kcal mol^-1^ (MAE) (Supplementary Fig. 2). These results demonstrate that the newly derived dihedral parameters improve the agreement with QM-derived energies and, thus, should be better suited to reproduce the conformational energies of the investigated cAMP derivatives.

## Supplementary Figures


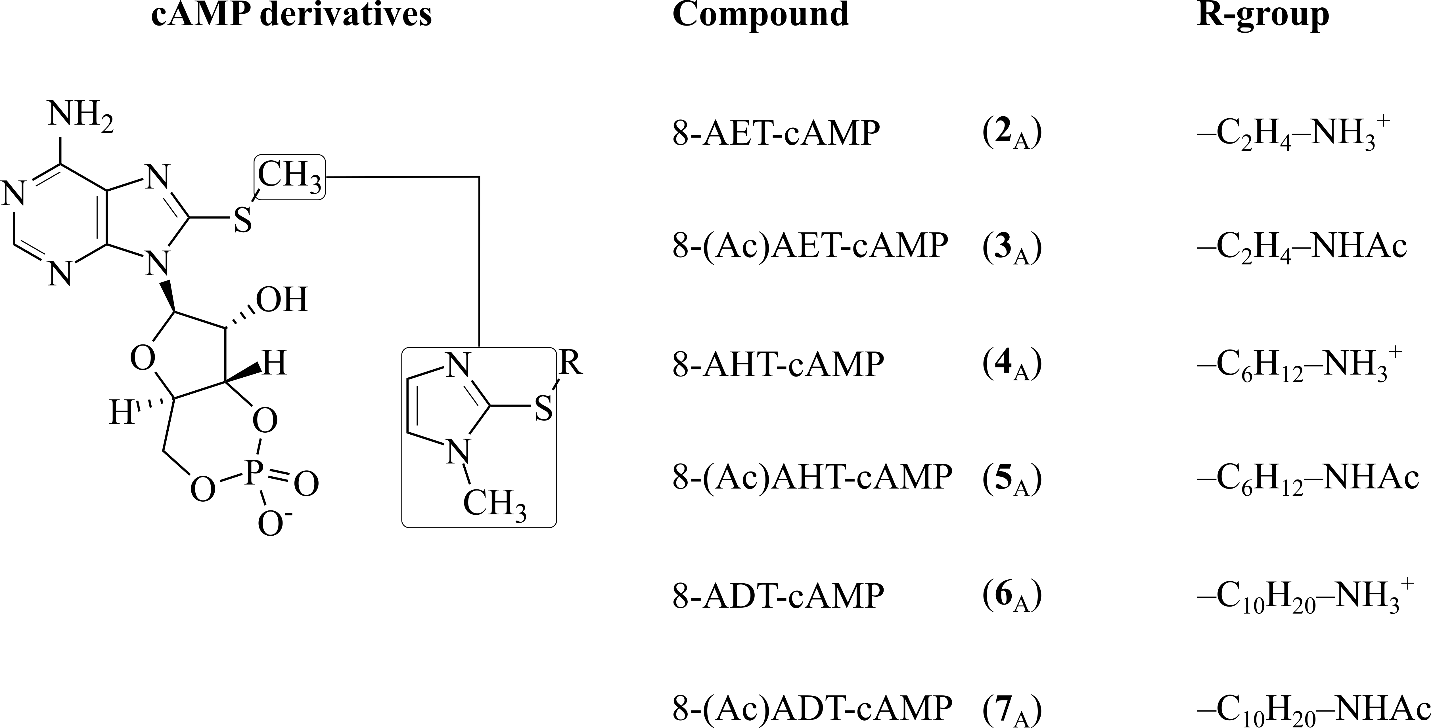


**Supplementary Figure 1:** Ligand parameterization scheme. Groups for which inter-molecular charge constraints were applied are indicated by rounded boxes and a connecting line.


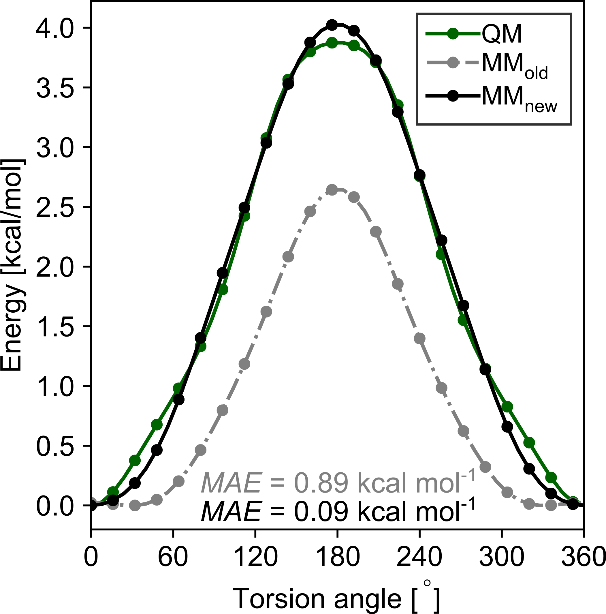


**Supplementary Figure 2:** Energy profile of the rotation about the na-cc-ss-c3 torsion in 2-(methylthio)-1H-imidazole. The new parameters (black) improve the agreement with the QM-derived energies (green) by, on average, 0.80 kcal mol^-1^ with respect to the parameters derived from the parmchk2 tool (grey).


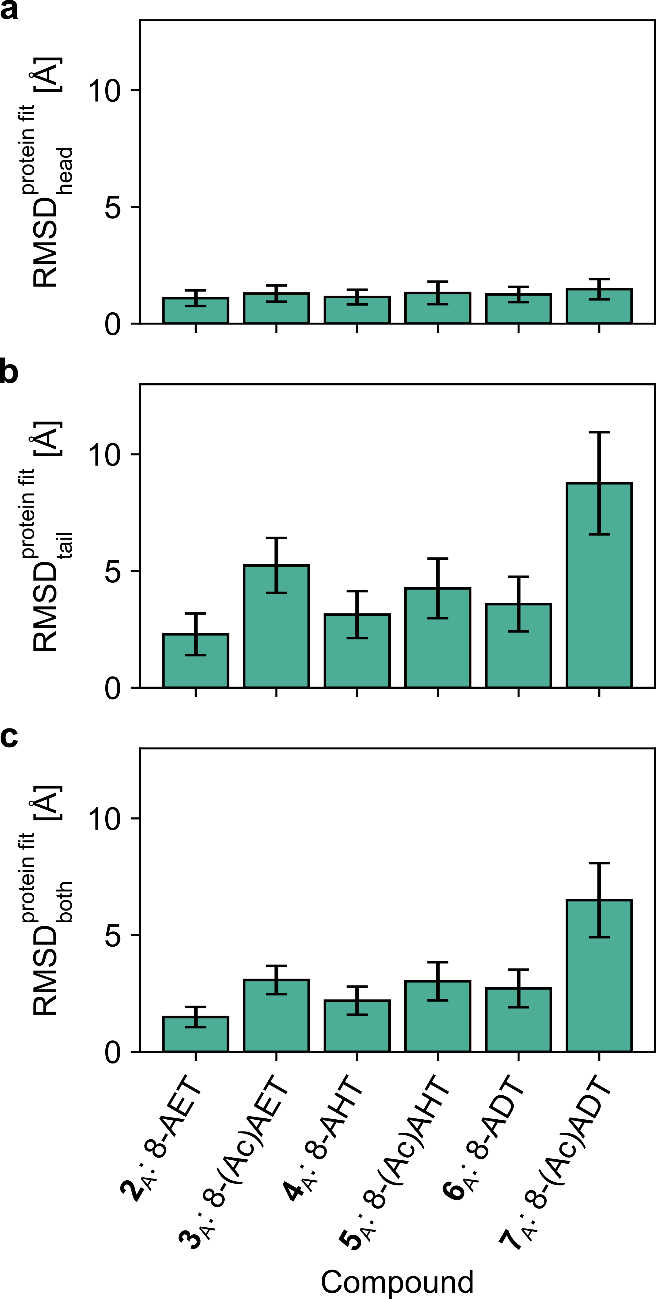


**Supplementary Figure 3:** Root-mean-square deviation (RMSD) of the atomic coordinates of the (a) head region, (b) tail region, and (c) complete ligand of the investigated cAMP derivatives after root-mean-square fitting of the protein backbone. RMSD values were calculated for all four ligands separately with respect to the starting structure and were averaged over the whole MD trajectory. Error bars display sample standard deviations.


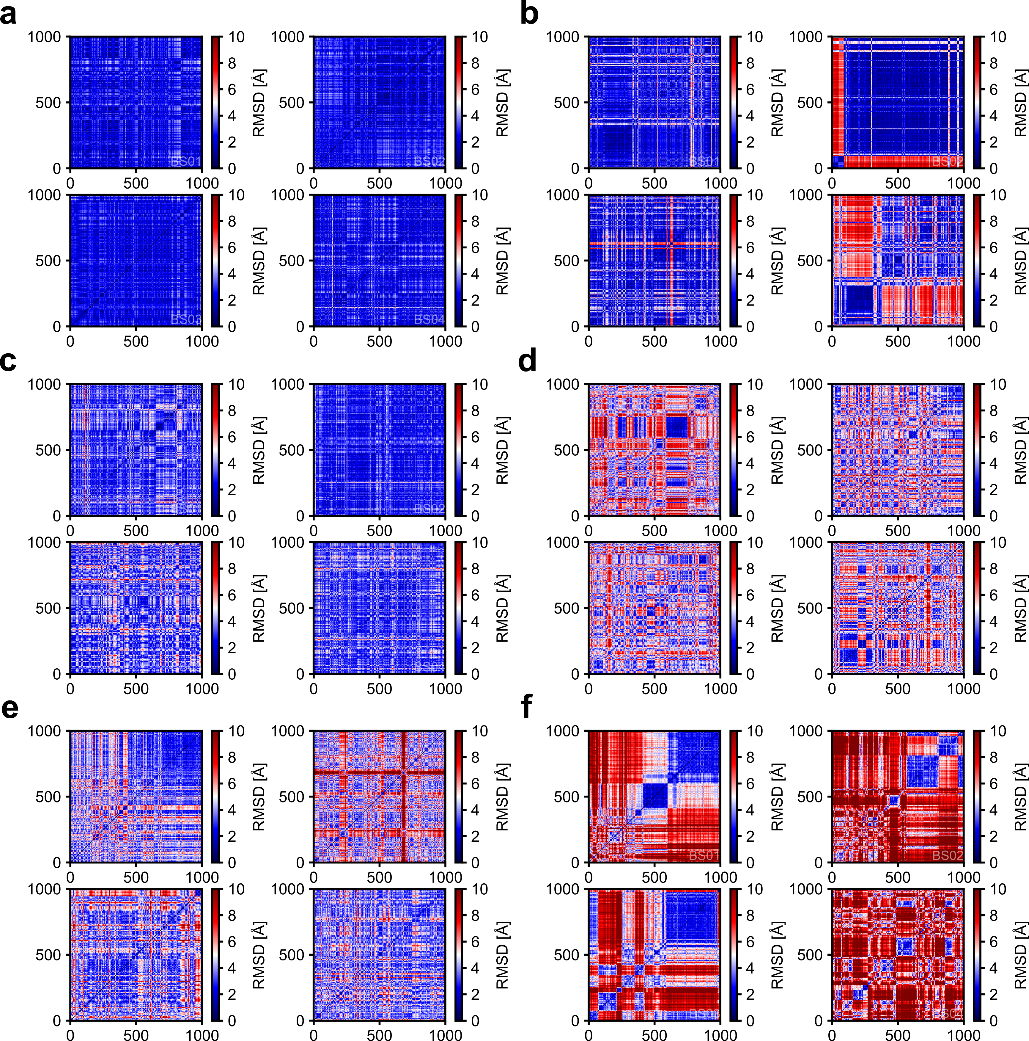


**Supplementary Figure 4:** Conformational heterogeneity of the ligand in complexes of rCNGA2 and cAMP-derivatives. Pairwise root-mean-square deviations (2D-RMSD) of the atomic coordinates of the tail region of the investigated cAMP-derivatives after root-mean-square fitting of the core/head region are color-coded from blue (RMSD = 0 Å) over white (RMSD = 5 Å) to red (RMSD = 10 Å). (a) 8-AET-cAMP (**2**_A_), (b) 8-(Ac)AET-cAMP (**3**_A_), (c) 8-AHT-cAMP (**4**_A_), (d) 8-(Ac)AHT-cAMP (**5**_A_), (e) 8-ADT-cAMP (**6**_A_), (f) 8-(Ac)ADT-cAMP (**7**_A_). Each of the four subpanels represents one binding site (BS01-BS04).


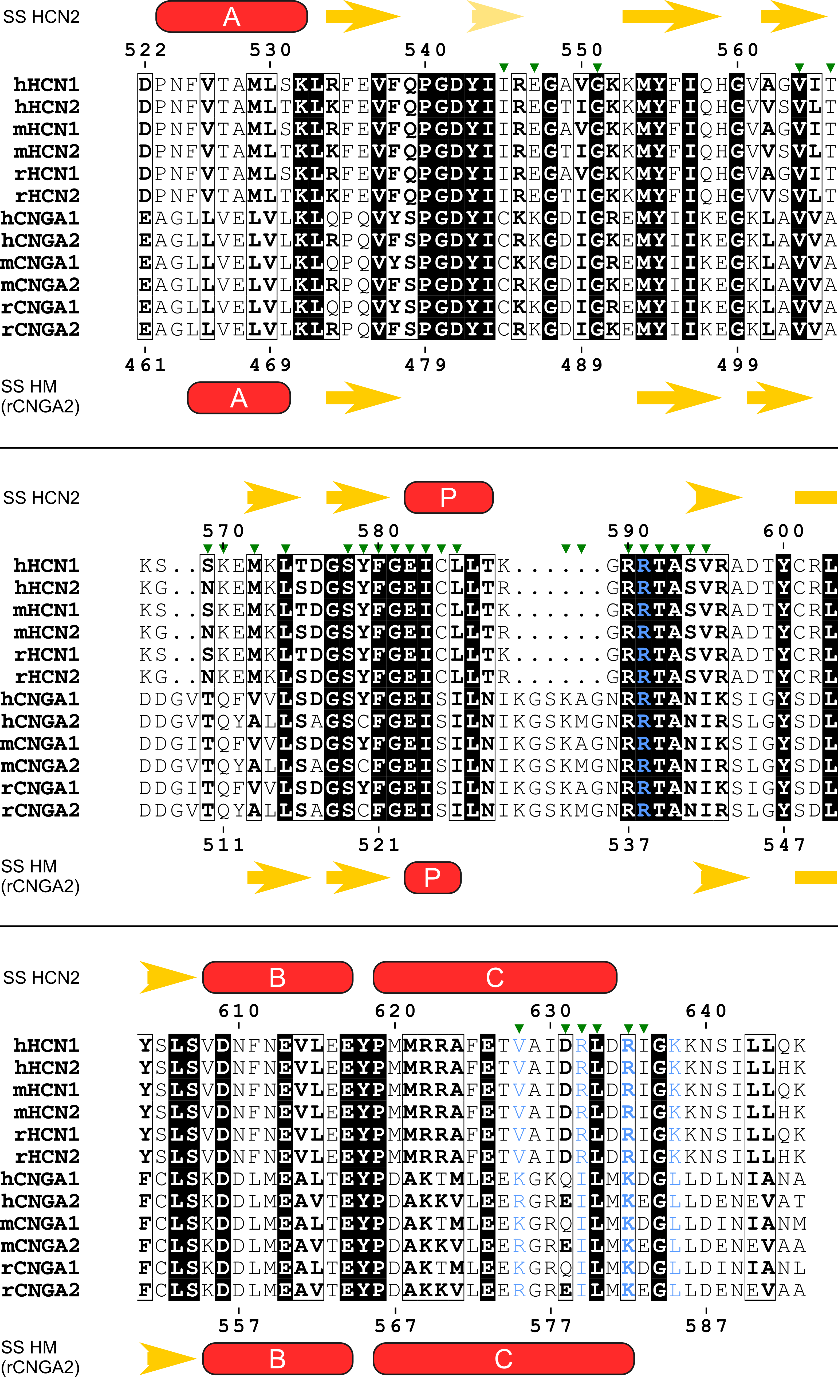


**Supplementary Figure 5:** **Sequence alignment of human, mouse, and rat HCN1/2 and CNGA1/2.** Top residue numbers correspond to HCN1/2, bottom residue numbers to rCNGA2. Secondary structure elements are indicated as red rods (helices) and orange arrows (sheets). Residues within 5 Å of the cNMPs are highlighted with green triangles. Identical regions are highlighted with white letters on black background, similar regions are highlighted with a surrounding box. Residues highlighted in blue have been described in other studies as important for cNMP binding in HCN2 channels^8^.

## Supplementary Tables

**Supplementary Table 1. Effects of cNMP derivatives on homotetrameric CNGA2 and heterotetrameric CNGA2:CNGA4:CNGB1b channels (a-d) and on homotetrameric HCN2 channels (e).** The table summarizes the *EC*_50_ values, Hill coefficients *H* (mean ± SEM), and number of experiments, n. * indicates that **2**_A_ acted as a partial agonist on CNGA2 channels. ** indicates that **6**_A_ evoked an unusual inhibition at concentrations > 2 µM in CNGA2:CNGA4:CNGB1b channels.

| 1. Effects of cGMP derivatives on homotetrameric CNGA2 channels | | | | | | | | |
| --- | --- | --- | --- | --- | --- | --- | --- | --- |
| **No. of compound** | **Chain type** | ***EC*_50_** | | | ***H*** | | | **n** |
| **1_G_** | cGMP | 1.8 | ± | 0.1 | 2.6 | ± | 0.04 | 16 |
| **2_G_** | 8-AET- | 0.83 | ± | 0.05 | 2.4 | ± | 0.29 | 14 |
| **3_G_** | 8-(Ac)AET- | 0.17 | ± | 0.03 | 1.8 | ± | 0.24 | 5 |
| **4_G_** | 8-AHT- | 0.09 | ± | 0.02 | 2.5 | ± | 0.54 | 5 |
| **5_G_** | 8-(Ac)AHT- | 0.08 | ± | 0.004 | 2.7 | ± | 0.14 | 10 |
| **6_G_** | 8-ADT- | 0.06 | ± | 0.006 | 1.8 | ± | 0.09 | 7 |
| **7_G_** | 8-(Ac)ADT- | 0.05 | ± | 0.003 | 2.2 | ± | 0.11 | 8 |
| **8_G_** | 8-(Ac)APT- | 0.55 | ± | 0.04 | 2.7 | ± | 0.25 | 12 |
| **9_G_** | 8-(Ac)APET- | 0.52 | ± | 0.03 | 2.3 | ± | 0.13 | 11 |
| **10_G_** | 8-(Ac)AGET- | 0.34 | ± | 0.01 | 2.6 | ± | 0.08 | 12 |
| **11_G_** | 8-(Ac)AGGET- | 0.33 | ± | 0.02 | 2.4 | ± | 0.11 | 11 |
|  | | | | | | | | |
| 1. Effects of cAMP derivatives on homotetrameric CNGA2 channels | | | | | | | | |
| **No. of compound** | **Chain type** | ***EC*_50_** | | | ***H*** | | | **n** |
| **1_A_** | cAMP | 54.8 | ± | 6.0 | 2.2 | ± | 0.04 | 4 |
| **2_A_** | 8-AET- | 128.3 | ± | 20.4* | 1.8 | ± | 0.17 | 14 |
| **3_A_** | 8-(Ac)AET- | 9.9 | ± | 0.8 | 2.3 | ± | 0.11 | 9 |
| **4_A_** | 8-AHT- | 3.1 | ± | 0.3 | 2.3 | ± | 0.12 | 10 |
| **5_A_** | 8-(Ac)AHT- | 1.4 | ± | 0.2 | 3.1 | ± | 0.28 | 8 |
| **6_A_** | 8-ADT- | 0.63 | ± | 0.06 | 2.3 | ± | 0.18 | 7 |
| **7_A_** | 8-(Ac)ADT- | 2.3 | ± | 0.4 | 2.4 | ± | 0.23 | 14 |
| **8_A_** | 8-(Ac)APT- | 24.9 | ± | 1.3 | 2.4 | ± | 0.11 | 7 |
| **9_A_** | 8-(Ac)APET- | 46.2 | ± | 6.9 | 2.3 | ± | 0.09 | 8 |
| **10_A_** | 8-(Ac)AGET- | 34.2 | ± | 4.5 | 2.1 | ± | 0.22 | 10 |
| **11_A_** | 8-(Ac)AGGET- | 29.37 | ± | 3.1 | 1.9 | ± | 0.11 | 7 |
|  | | | | | | | | |

| 1. Effects of cGMP derivatives on heterotetrameric CNGA2:CNGA4:CNGB1b channels | | | | | | | | |
| --- | --- | --- | --- | --- | --- | --- | --- | --- |
| **No. of compound** | **Chain type** | ***EC*_50_** | | | ***H*** | | | **n** |
| **1_G_** | cGMP | 1.3 | ± | 0.1 | 2.5 | ± | 0.28 | 8 |
| **2_G_** | 8-AET- | 1.5 | ± | 0.2 | 1.8 | ± | 0.21 | 5 |
| **3_G_** | 8-(Ac)AET- | 0.28 | ± | 0.02 | 2.0 | ± | 0.10 | 6 |
| **4_G_** | 8-AHT- | 0.62 | ± | 0.17 | 1.3 | ± | 0.14 | 5 |
| **5_G_** | 8-(Ac)AHT- | 0.23 | ± | 0.01 | 2.0 | ± | 0.09 | 10 |
| **6_G_** | 8-ADT- | 0.30 | ± | 0.02 | 1.6 | ± | 0.10 | 6 |
| **7_G_** | 8-(Ac)ADT- | 0.14 | ± | 0.02 | 1.9 | ± | 0.12 | 7 |
| **8_G_** | 8-(Ac)APT- | 1.1 | ± | 0.1 | 1.6 | ± | 0.07 | 8 |
| **9_G_** | 8-(Ac)APET- | 1.1 | ± | 0.1 | 2.0 | ± | 0.18 | 9 |
| **10_G_** | 8-(Ac)AGET- | 0.63 | ± | 0.03 | 1.9 | ± | 0.07 | 9 |
| **11_G_** | 8-(Ac)AGGET- | 1.0 | ± | 0.2 | 1.7 | ± | 0.12 | 10 |
|  | | | | | | | | |
| 1. Effects of cAMP derivatives on heterotetrameric CNGA2:CNGA4:CNGB1b channels | | | | | | | | |
| **No. of compound** | **Chain type** | ***EC*_50_** | | | ***H*** | | | **n** |
| **1_A_** | cAMP | 5.0 | ± | 0.3 | 2.1 | ± | 0.08 | 7 |
| **2_A_** | 8-AET- | 13.3 | ± | 0.7 | 1,0 | ± | 0.02 | 8 |
| **3_A_** | 8-(Ac)AET- | 4.1 | ± | 0.6 | 1.8 | ± | 0.37 | 5 |
| **4_A_** | 8-AHT- | 1.4 | ± | 0.1 | 2.5 | ± | 0.06 | 6 |
| **5_A_** | 8-(Ac)AHT- | 0.95 | ± | 0.06 | 2.5 | ± | 0.19 | 5 |
| **6_A_** | 8-ADT- | 0.29 | ± | 0.04** | 3.9 | ± | 1.04 | 9 |
| **7_A_** | 8-(Ac)ADT- | 0.47 | ± | 0.06 | 2.7 | ± | 0.20 | 10 |
| **8_A_** | 8-(Ac)APT- | 6.0 | ± | 0.4 | 2.2 | ± | 0.10 | 10 |
| **9_A_** | 8-(Ac)APET- | 7.1 | ± | 0.3 | 2.2 | ± | 0.13 | 7 |
| **10_A_** | 8-(Ac)AGET- | 6.6 | ± | 0.4 | 2.2 | ± | 0.14 | 8 |
| **11_A_** | 8-(Ac)AGGET- | 5.1 | ± | 0.6 | 2.5 | ± | 0.23 | 6 |
|  | | | | | | | | |
| 1. Effects of cAMP derivatives on homotetrameric HCN2 channels | | | | | | | | |
| **No. of compound** | **Chain type** | ***EC*_50_** | | | ***H*** | | | **n** |
| **1_A_** | cAMP | 28.8 | ± | 3.7 | 1.3 | ± | 0.13 | 9 |
| **3_A_** | 8-(Ac)AET- | 70.6 | ± | 9.2 | 1.7 | ± | 0.61 | 7 |
| **5_A_** | 8-(Ac)AHT- | 19.7 | ± | 1.9 | 0.8 | ± | 0.07 | 8 |
| **7_A_** | 8-(Ac)ADT- | 14.6 | ± | 3.4 | 1.5 | ± | 0.27 | 9 |
| **8_A_** | 8-(Ac)APT- | 144.3 | ± | 15.9 | 0.9 | ± | 0.11 | 6 |
| **10_A_** | 8-(Ac)AGET- | 96.7 | ± | 23.7 | 1.5 | ± | 0.45 | 6 |

**Supplementary Table 2. Analytical details of the synthesized compounds.**

| **No.** | **Short Name** | **Purity**  **(area %)** | **HPLC**^a^  **(Rt in min)** | **MS found**  **(m/z)** | **TLC**^b^  **(R_f_-values)** |
| --- | --- | --- | --- | --- | --- |
| **2**_A_ | 8-AET-cAMP | >99 | 11.2^c^ | (M-H)^-^ 403.06  (M+H)^+^ 405.07 | 0.18 |
| **2**_G_ | 8-AET-cGMP | >99 | 12.4^c^ | (M-H)^-^ 419.05  (M+H)^+^ 421.07 | 0.28 |
| **3**_A_ | 8-(Ac)AET-cAMP | >99 | 19.5^c^ | (M-H)^-^ 445.07 | 0.40 |
| **3**_G_ | 8-(Ac)AET-cGMP | >99 | 19.9^c^ | (M-H)^-^ 461.07 | 0.48 |
| **4**_A_ | 8-AHT-cAMP | >99 | 19.8^e^ | (M-H)^-^ 459.12  (M+Na)^+^ 483.12 | 0.24 |
| **4**_G_ | 8-AHT-cGMP | >99 | 21.9 | (M-H)^-^ 475.12  (M+Na)^+^ 499.11 | 0.34 |
| **5**_A_ | 8-(Ac)AHT-cAMP | >99 | 34.8 | (M-H)^-^ 501.13  (M+H)^+^ 503.15 | 0.65 |
| **5**_G_ | 8-(Ac)AHT-cGMP | 97 | 32.1 | (M-H)^-^ 517.13 | 0.55 |
| **6**_A_ | 8-ADT-cAMP | 97 | 15.0^d,e^ | (M-H)^-^ 515.19  (M+Na)^+^ 539.18 | 0.60 |
| **6**_G_ | 8-ADT-cGMP | >99 | 18.1^d^ | (M-H)^-^ 531.18  (M+Na)^+^ 555.18 | 0.60 |
| **7**_A_ | 8-(Ac)ADT-cAMP | >99 | 25.5^d^ | (M-H)^-^ 557.19  (M+H)^+^ 559.21 | 0,64 |
| **7**_G_ | 8-(Ac)ADT-cGMP | 95 | 28.4^d,e^ | (M-H)^-^ 573.19  (M+H)^+^ 575.2 | 0.68 |
| **8**_A_ | 8-(Ac)APT-cAMP | >99 | 24.6 | (M-H)^-^ 553.13  (M+H)^+^ 535.14 | 0.29 |
| **8**_G_ | 8-(Ac)APT-cGMP | >99 | 23.5 | (M-H)^-^ 549.12  (M+H)^+^ 551.13 | 0.46 |
| **9**_A_ | 8-(Ac)APET-cAMP | 98 | 22.9 | (M-H)^-^ 590.14  (M+H)^+^ 592.16 | 0.21 |
| **9**_G_ | 8-(Ac)APET-cGMP | >95 | 22.0 | (M-H)^-^ 606.14  (M+H)^+^ 608.15 | 0.32 |
| **10**_A_ | 8-(Ac)AGET-cAMP | >99 | 16.0 | (M-H)^-^ 502.09  (M+H)^+^ 504.11 | 0.30 |
| **10**_G_ | 8-(Ac)AGET-cGMP | >99 | 15.7 | (M-H)^-^ 518.09  (M+H)^+^ 520.10 | 0.35 |
| **11**_A_ | 8-(Ac)AGGET-cAMP | >99 | 16.7 | (M-H)^-^ 559.11  (M+H)^+^ 561.13 | 0.20 |
| **11**_G_ | 8-(Ac)AGGET-cGMP | >99 | 16.6 | (M-H)^-^ 575.11  (M+H)^+^ 577.19 | 0.26 |

Solvent systems:

^a^ standard conditions for analytical HPLC: linear water (A)-methanol (B) gradient with 0.1 % formic acid at a flow rate of 1 ml/min (increase of 1 % B/min), 30°C, monitored at 254 nm, start at 1 % B (^c^ start at 0 % B, 25°C; ^d^start at 30 % B). All compounds were purified by semipreparative HPLC with a water-methanol gradient containing 0.1 % formic acid; ^e^ in these rare cases the eluent for semipreparative HPLC contained 5 mM ammonium acetate (pH 5) in the aqueous phase.

^b^ TLC n-butanol/acetic acid/H_2_O: 5/3/2(v/v/v).

## References

1 Brown, R. L., Bert, R. J., Evans, F. E. & Karpen, J. W. Activation of retinal rod cGMP-gated channels: what makes for an effective 8-substituted derivative of cGMP? *Biochemistry* **32**, 10089-10095 (1993).

2 Caretta, A., Cavaggioni, A. & Sorbi, R. T. Binding Stoichiometry of a Fluorescent Cgmp Analog to Membranes of Retinal Rod Outer Segments. *Eur J Biochem* **153**, 49-53, doi:DOI 10.1111/j.1432-1033.1985.tb09265.x (1985).

3 Bergmann, E. D. & Migron, Y. Preparation of Olefinic Derivatives of Phthalimide and Their Use as Precursors for Homoallylic Amines. *Org Prep Proced Int* **8**, 75-80, doi:Doi 10.1080/00304947609355592 (1976).

4 Li, M. *et al.* Structure of a eukaryotic cyclic-nucleotide-gated channel. *Nature* **542**, 60-65, doi:10.1038/nature20819 (2017).

5 Biasini, M. *et al.* SWISS-MODEL: modelling protein tertiary and quaternary structure using evolutionary information. *Nucleic Acids Res* **42**, W252-258, doi:10.1093/nar/gku340 (2014).

6 Chen, V. B. *et al.* MolProbity: all-atom structure validation for macromolecular crystallography. *Acta Crystallogr D Biol Crystallogr* **66**, 12-21, doi:10.1107/S0907444909042073 (2010).

7 Chen, V. B., Davis, I. W. & Richardson, D. C. KING (Kinemage, Next Generation): a versatile interactive molecular and scientific visualization program. *Protein Sci* **18**, 2403-2409, doi:10.1002/pro.250 (2009).

8 Zagotta, W. N. *et al.* Structural basis for modulation and agonist specificity of HCN pacemaker channels. *Nature* **425**, 200-205, doi:10.1038/nature01922 (2003).

9 Schrödinger, LLC. *Schrödinger Release 2017-1: Maestro* (2017).

10 Frisch, M. J. *et al.* (Wallingford CT, 2009).

11 Vanquelef, E. *et al.* R.E.D. Server: a web service for deriving RESP and ESP charges and building force field libraries for new molecules and molecular fragments. *Nucleic Acids Res* **39**, W511-517, doi:10.1093/nar/gkr288 (2011).

12 Dupradeau, F.-Y. *et al.* The R.E.D. tools: advances in RESP and ESP charge derivation and force field library building. *Phys Chem Chem Phys* **12**, 7821-7839, doi:10.1039/c0cp00111b (2010).

13 Wang, J., Cieplak, P. & Kollman, P. A. How well does a restrained electrostatic potential (RESP) model perform in calculating conformational energies of organic and biological molecules? *J Comput Chem* **21**, 1049-1074, doi:Doi 10.1002/1096-987x(200009)21:12<1049::Aid-Jcc3>3.3.Co;2-6 (2000).

14 Cornell, W. D. *et al.* A Second Generation Force Field for the Simulation of Proteins, Nucleic Acids, and Organic Molecules. *J Am Chem Soc* **117**, 5179-5197, doi:10.1021/ja00124a002 (1995).

15 Hornak, V. *et al.* Comparison of Multiple Amber Force Fields and Development of Improved Protein Backbone Parameters. *Proteins* **65**, 712-725, doi:10.1002/prot.21123 (2006).

16 Case, D. A. *et al.* *AMBER 2017* (University of California, San Francisco, 2017).

17 Wang, J., Wolf, R. M., Caldwell, J. W., Kollman, P. A. & Case, D. A. Development and testing of a general amber force field. *J Comput Chem* **25**, 1157-1174, doi:10.1002/jcc.20035 (2004).

18 Wang, J., Wang, W., Kollman, P. A. & Case, D. A. Automatic atom type and bond type perception in molecular mechanical calculations. *J Mol Graph Model* **25**, 247-260, doi:10.1016/j.jmgm.2005.12.005 (2006).

19 Friesner, R. A. *et al.* Extra precision glide: docking and scoring incorporating a model of hydrophobic enclosure for protein-ligand complexes. *J Med Chem* **49**, 6177-6196, doi:10.1021/jm051256o (2006).

20 Rostkowski, M., Olsson, M. H., Sondergaard, C. R. & Jensen, J. H. Graphical analysis of pH-dependent properties of proteins predicted using PROPKA. *BMC Struct Biol* **11**, 6, doi:10.1186/1472-6807-11-6 (2011).

21 Olsson, M. H. M., Søndergaard, C. R., Rostkowski, M. & Jensen, J. H. PROPKA3: Consistent Treatment of Internal and Surface Residues in Empirical pKa Predictions. *J Chem Theory Comput* **7**, 525-537, doi:10.1021/ct100578z (2011).

22 Lomize, M. A., Pogozheva, I. D., Joo, H., Mosberg, H. I. & Lomize, A. L. OPM database and PPM web server: resources for positioning of proteins in membranes. *Nucleic Acids Res* **40**, D370-376, doi:10.1093/nar/gkr703 (2012).

23 Jo, S., Kim, T., Iyer, V. G. & Im, W. CHARMM-GUI: a web-based graphical user interface for CHARMM. *J Comput Chem* **29**, 1859-1865, doi:10.1002/jcc.20945 (2008).

24 Maier, J. A. *et al.* ff14SB: Improving the Accuracy of Protein Side Chain and Backbone Parameters from ff99SB. *J Chem Theory Comput* **11**, 3696-3713, doi:10.1021/acs.jctc.5b00255 (2015).

25 Dickson, C. J. *et al.* Lipid14: The Amber Lipid Force Field. *J Chem Theory Comput* **10**, 865-879, doi:10.1021/ct4010307 (2014).

26 Le Grand, S., Götz, A. W. & Walker, R. C. SPFP: Speed without compromise-A mixed precision model for GPU accelerated molecular dynamics simulations. *Comput Phys Commun* **184**, 374-380, doi:10.1016/j.cpc.2012.09.022 (2013).

27 Pastor, R. W., Brooks, B. R. & Szabo, A. An Analysis of the Accuracy of Langevin and Molecular-Dynamics Algorithms. *Mol Phys* **65**, 1409-1419, doi:Doi 10.1080/00268978800101881 (1988).

28 Ryckaert, J.-P., Ciccotti, G. & Berendsen, H. J. C. Numerical-Integration of Cartesian Equations of Motion of a System with Constraints - Molecular-Dynamics of N-Alkanes. *J Comput Phys* **23**, 327-341, doi:Doi 10.1016/0021-9991(77)90098-5 (1977).

29 Darden, T., York, D. & Pedersen, L. Particle Mesh Ewald - an N⋅log(N) Method for Ewald Sums in Large Systems. *J Chem Phys* **98**, 10089-10092, doi:Doi 10.1063/1.464397 (1993).

30 Roe, D. R. & Cheatham, T. E., III. PTRAJ and CPPTRAJ: Software for Processing and Analysis of Molecular Dynamics Trajectory Data. *Journal of Chemical Theory and Computation* **9**, 3084-3095, doi:10.1021/Ct400341p (2013).

31 Wang, C., Greene, D. A., Xiao, L., Qi, R. & Luo, R. Recent Developments and Applications of the MMPBSA Method. *Front Mol Biosci* **4**, 87, doi:10.3389/fmolb.2017.00087 (2017).

32 Gohlke, H. & Case, D. A. Converging free energy estimates: MM-PB(GB)SA studies on the protein-protein complex Ras-Raf. *J Comput Chem* **25**, 238-250, doi:10.1002/jcc.10379 (2004).

33 Srinivasan, J., Cheatham, T. E., III, Cieplak, P., Kollman, P. A. & Case, D. A. Continuum solvent studies of the stability of DNA, RNA, and phosphoramidate - DNA helices. *J Am Chem Soc* **120**, 9401-9409, doi:DOI 10.1021/ja981844+ (1998).

34 Miller, B. R., III *et al.* MMPBSA.py: An Efficient Program for End-State Free Energy Calculations. *J Chem Theory Comput* **8**, 3314-3321, doi:10.1021/ct300418h (2012).

35 Tan, C., Yang, L. & Luo, R. How well does Poisson-Boltzmann implicit solvent agree with explicit solvent? A quantitative analysis. *J Phys Chem B* **110**, 18680-18687, doi:10.1021/jp063479b (2006).

36 Tsui, V. & Case, D. A. Theory and applications of the generalized Born solvation model in macromolecular simulations. *Biopolymers* **56**, 275-291, doi:10.1002/1097-0282(2000)56:4<275::AID-BIP10024>3.0.CO;2-E (2000).

37 Tan, C., Tan, Y.-H. & Luo, R. Implicit nonpolar solvent models. *J Phys Chem B* **111**, 12263-12274, doi:10.1021/jp073399n (2007).

38 Hawkins, G. D., Cramer, C. J. & Truhlar, D. G. Pairwise Solute Descreening of Solute Charges from a Dielectric Medium. *Chem Phys Lett* **246**, 122-129, doi:Doi 10.1016/0009-2614(95)01082-K (1995).

39 Weis, A., Katebzadeh, K., Söderhjelm, P., Nilsson, I. & Ryde, U. Ligand affinities predicted with the MM/PBSA method: dependence on the simulation method and the force field. *J Med Chem* **49**, 6596-6606, doi:10.1021/jm0608210 (2006).

40 Page, C. S. & Bates, P. A. Can MM-PBSA calculations predict the specificities of protein kinase inhibitors? *J Comput Chem* **27**, 1990-2007, doi:10.1002/jcc.20534 (2006).

41 Genheden, S., Kuhn, O., Mikulskis, P., Hoffmann, D. & Ryde, U. The normal-mode entropy in the MM/GBSA method: effect of system truncation, buffer region, and dielectric constant. *J Chem Inf Model* **52**, 2079-2088, doi:10.1021/ci3001919 (2012).
